# An antibody with Fab-constant domains exchanged for a pair of C_H_3 domains

**DOI:** 10.1371/journal.pone.0195442

**Published:** 2018-04-09

**Authors:** Gordana Wozniak-Knopp, Gerhard Stadlmayr, Jan Walther Perthold, Katharina Stadlbauer, Mathias Gotsmy, Stefan Becker, Florian Rüker

**Affiliations:** 1 Christian Doppler Laboratory for Innovative Immunotherapeutics, Department of Biotechnology, University of Natural Resources and Life Sciences (BOKU), Vienna, Austria; 2 Institute of Molecular Modeling and Simulation, Department of Material Sciences, University of Natural Resources and Life Sciences (BOKU), Vienna, Austria; 3 Protein Engineering and Antibody Technologies, Merck KGaA, Darmstadt, Germany; University of Colorado Anschutz Medical Campus, UNITED STATES

## Abstract

We have designed a complete antibody-like construct where the C_H_1 and C_κ_ domains are exchanged for a pair of the C_H_3 domains and efficient pairing of the heavy and light variable domain is achieved using “Knobs-into-Holes” strategy. This construct, composed of only naturally occurring immunoglobulin sequences without artificial linkers, expressed at a high level in mammalian cells, however exhibited low solubility. Rational mutagenesis aimed at the amino acid residues located at the interface of the variable domains and the exchanged C_H_3 domains was applied to improve the biophysical properties of the molecule. The domain-exchanged construct, including variable domains of the HER2/neu specific antibody trastuzumab, was able to bind to the surface of the strongly HER2/neu positive cell line SK-BR3 4-fold weaker than trastuzumab, but could nevertheless incite a more potent response in an antibody-dependent cell cytotoxicity (ADCC) reporter assay with FcγRIIIa-overexpressing T-cells. This could be explained with a stronger binding to the FcγRIIIa. Importantly, the novel construct could mediate a specific ADCC effect with natural killer cells similar to the parental antibody.

## Introduction

The IgG molecule is a globular protein composed of two identical heavy chains, folded into four domains, and two identical light chains, folded into two domains. Two of these units are Fab fragments composed of each V_H_, C_H_1, V_L_ and C_L_ domain, and the third unit is the Fc fragment, a homodimer of two C_H_2 and two C_H_3 domains. Domain members of the immunoglobulin superfamily are characterized by their β-sandwich topology comprising two β-sheets, each forming a Greek-key folding motif [1]. Despite the remarkable variation in sequence and function, the members of the immunoglobulin superfamily exhibit few distinct variable folding sets [2]. C-class immunoglobulin domains are of particular interest because of their high stability and the potential to elicit effector functions. Although mostly the variable domains are engaged with determining the antibody specificity and the Fc fragment with triggering the phenomena related to the biological effect of an antibody such as lysis or phagocytosis of target cells via binding to the different Fc receptor molecules, the efforts to functionalize constant domains of antibodies to harbor newly designed antigen binding sites and simultaneously incite effector functions have already been successful [3, 4] and even proceeded to the point of clinical relevance [5]. Several successful improvements of the stability of constant domains have been reported [6–8], which illustrates their potential for further functionalization of antibodies.

There is little published data on how the Fab constant domains can contribute to the conformational stability and subsequently antigen binding mediated by variable domains. It has been shown that both C_H_1 and C_L_ domains are required for stabilization and only the disulfide bridged C_H_1/C_L_ pair has reached the stability of the most stable variable domains [9]. A systematic study aimed at rational stability engineering of a mouse IgG1 Fab fragment has exploited (1) the mutagenesis of the flexible loop connecting the first two β-strands in the C_H_1 domain, (2) introduction of hydrophobic residues in the hydrophobic core regions of C_H_1 and C_L_ and (3) introduction of hydrophobic residues into the two largest hydrophobic cavities at the interface of the C_H_1 and C_L_-domains and the final strategy proved beneficial for both the thermal stability and the expression level of the antibody fragment [10].

In the antibodies the movement of the V_H_-V_L_ dimer relative to the C_H_1-C_L_ dimer involves the interactions of three V_H_ and two C_H_1 residues that form the molecular equivalent of a ball-and-socket joint [11], and similar features have been described in the C_H_2-C_H_3 interface [12]. Although the C_H_2-C_H_3 interface is tightly packed, water structure can be observed in the high resolution structures of Fc fragments of different classes of IgGs. The C_H_2-C_H_3 domain interface is conserved across all antibody isotypes and stabilized with 2 salt bridges, 2 hydrogen bonds and further via interactions over bridging water molecules [13]. There is however no systematic generalization that could correlate the relative position of the C_H_2 to the C_H_3 domains and the functional differences in the corresponding antibodies, such as Fc receptor binding [13].

In naturally occurring antibodies, heavy and light chain of the Fab fragment are usually connected with a disulfide bond that is structurally located at the bottom of the constant domains. The exact position of the cysteine and the torsion angle hence formed between the C-termini of the C_H_1 domain and the C_L_ domain differ among the antibodies and this appears to influence strongly their stability as well as their ability to bind to Fcγ receptors (FcγR), as illustrated by the example of deletion of the naturally occurring serine residue from the sequence of the antibody chains of the lambda class, which improved their resistance to chemical degradation as well as their potency in target cell lysis via antibody-dependent cell cytotoxicity (ADCC) [14].

The homology in the structure of C_H_1/C_κ_ heterodimer and the C_H_3/C_H_3 domain pair has prompted us to attempt to exchange the pair of the constant domains of the variable region of trastuzumab (Herceptin®) with a pair of C_H_3 domains. The region between the variable and constant domains was composed only of their naturally present amino acids. To improve the yield of heterodimeric species, the minimal mutational variant of “Knobs-into-Holes” (Thr366Tyr/Tyr407Thr) [15] or ZW1 heterodimerization mutations [16], assigning a higher thermostability to the construct, were introduced. The 3 C-terminal amino acid residues of the newly introduced C_H_3 domains were replaced for the Fab-inherent C-terminal sequences that enable the formation of an interdomain cysteine bond analogous to one connecting C_H_1 and C_κ_ domains [17]. This modification led to an antibody-like molecular species (TRA-C_H_3_KiH_ or TRA-C_H_3_ZW1_) that was in over 95% homodimeric, however exhibited low solubility and a lower avidity to the cell-bound antigen HER2/neu than the parental antibody. As shown with analogously designed Fab-like constructs (TRA-Fab-C_H_3_KiH_ or TRA-Fab-C_H_3_ZW1_), monovalent antigen binding was less affected by the exchange of constant domains. Several point mutations, intended to optimize the novel interface between variable and constant domains, were tested for their influence on the thermal stability and the solubility of the initial construct. Although the binding of the domain-exchanged antibody to the strongly HER2/neu—positive cell line SK-BR3 was about 4-fold weaker than trastuzumab, it was surprisingly able to elicit a more potent response of FcγRIIIa-overexpressing T-cells in an ADCC reporter bioassay, which could be explained with its stronger binding to the FcγRIIIa. In an ADCC assay with natural killer cells from naïve donors, the domain-exchanged antibody was able to lyse the target cells to the same extent as the unmodified trastuzumab antibody.

## Materials and methods

### Design of the domain-exchanged antibody

**Fig 1:**
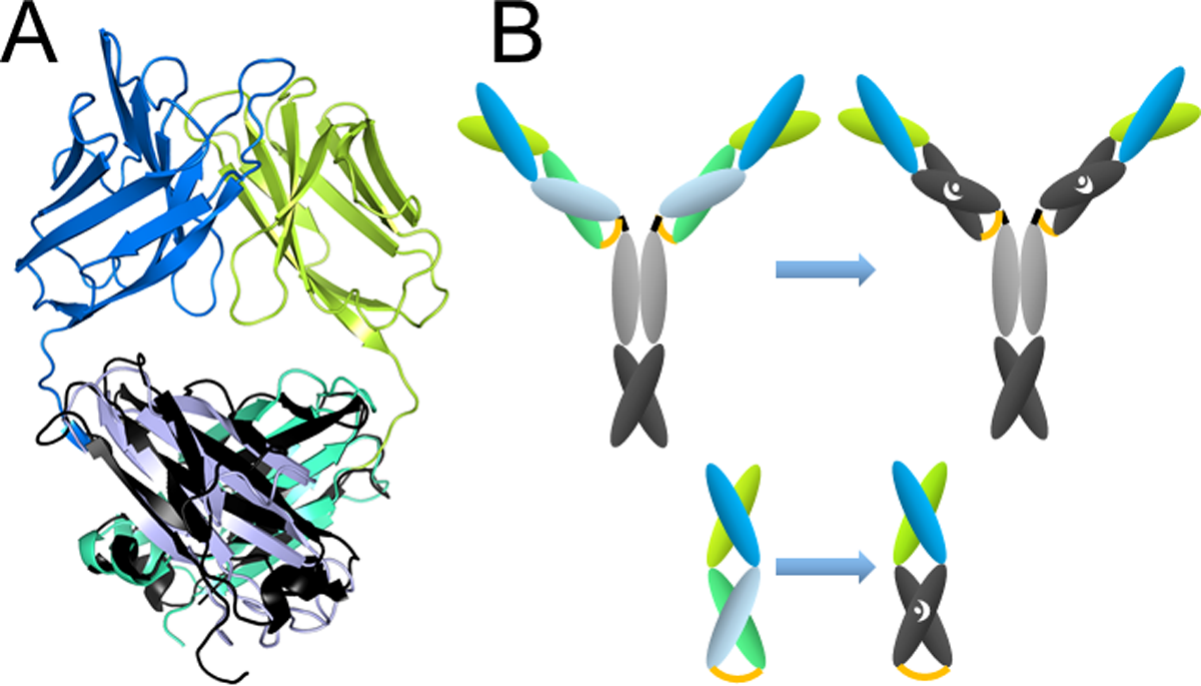
Graphical representation of the domain-exchanged antibody. (A) Superimposition of cartoon diagrams of structures of the 4D5 fragment (PDB: 1FVE) and C_H_3 domains (PDB: 1OQO). The figure was prepared using PyMol Molecular Graphics System. (B) Schematic of domain-exchanged antibody and the analogous Fab-like fragment. V_H_: dark blue, C_H_1: light blue, V_κ_: lemon, C_κ_: teal, C_H_2: gray, C_H_3: black. Heterodimerization motif is indicated with white symbols dot and crescent for “Knob and Hole”. Cysteine bond connecting the novel C_H_3 domains is in orange.

The pair of the C_H_3-domains (source: PDB 1OQO) was superimposed with the coordinates of the trastuzumab Fab fragment (source: PDB 1FVE, chains A and B) and resulted in an all-atom RMSD (root-mean-square deviation) after outlier rejection of only 1.62 Å of the overlay of both structures (Fig 1A). This result indicates a high degree of structural similarity between the constant domains. For the heavy chain the superimposition suggested the best fit when the last residue of the V_H_ was Gly122 (EU numbering scheme) and the start of the C_H_3 domain was Glu345 (amino acid sequences of all constructs are listed in S1 Table). For the light chain the best fit was with Ala111 as the last residue of the V_κ_ and Glu345 as the start of the C_H_3 domain. The C-terminal residues of the C_H_3 domain (ProGlyLys) were modified to LysSerCys when fused with the V_H_ and to GlyGluCys when fused with the V_κ_, to allow the formation of an interdomain cysteine bond [17]. The amino acid sequence of the heavy chain of the construct continued into an unmodified hinge region, C_H_2 domain and C_H_3 domain sequence of the IgG1 (S1 Table). Efficient heterodimerization of the light and heavy chain was achieved by the introduction of the “Knob” mutation Thr366Tyr into V_H_-linked C_H_3 domain and the “Hole” mutation Tyr407Thr of the V_κ_-linked C_H_3 domain (Fig 1B). This constellation was favored because one could expect a relatively larger amount of undesired “Hole-Hole” homodimers comparing with “Knob-Knob” homodimers upon expression [15], which are purified with Protein A along with the desired heterodimeric species. This construct was named TRA-C_H_3_KiH_ and the analogous construct with the ZW1-heterodimerization mutations was named TRA-C_H_3_ZW1_.

### Optimization of the variable domain—C_H_3 interface

To improve biophysical characteristics such as thermostability and solubility of the domain-exchanged antibodies, an optimization of the newly created variable domain–C_H_3 interface was performed. To guide the selection of possible stabilizing mutations, a structural model of the TRA-C_H_3_KiH_ Fab part was constructed. The structure of a C_H_3-domain dimer (source: PDB 1OQO) was superimposed to the constant domains of the trastuzumab Fab fragment (source: PDB 1FVE, chains A and B) using the PyMOL Molecular Graphics System (Schrödinger, LLC.). The constant domains of the trastuzumab Fab were then removed and the variable domains connected to the C_H_3-domains according to the sequences given in S2 Table. The connecting residues were rotated slightly around their Phi/Psi dihedral angles to allow geometrically for the formation of the peptide bonds between the variable and C_H_3-domains. To remove possible high-energy conformations, especially in the regions of the newly created interface and of the residues connecting the variable and the constant domains, the constructed molecule was energy-minimized using the RepairPDB function of the FoldX software [18] (version 3.0b6). Potential mutations that could contribute to optimization of the novel variable and constant domain interface were identified by visual inspection of the modelled TRA-C_H_3_KiH_ Fab molecule and comparison to the variable domain–C_H_1-C_L_ interface. Models of the investigated mutants (shown in S1 Fig) were constructed from the structural model of the wild-type TRA-C_H_3_KiH_ Fab using the mutagenesis wizard of PyMOL, which selects side-chain rotamers of the mutated residues based on their occurrences in protein structures.

### Mutagenesis and production of the recombinant protein

The chains of the domain-exchanged mutants (amino acid sequences in S1 Table) were cloned as a fusion protein of V_H_- and V_κ_- with a C_H_3 domain into a pTT5 vector (CNRC). Knob-into-Hole and ZW1 mutations driving heterodimerization (S1 Table) as well as the interface stabilizing mutations (S2 Table) were introduced using Quikchange Lightning Site-Directed Mutagenesis kit (Agilent), exactly according to manufacturer’s instructions, using the oligonucleotides listed in S3 Table. HEK293-6E cells (CNRC) were transfected with the heavy and the light chain of the construct in 1:1 mass ratio and kept under 5% CO_2_ in humidified atmosphere at 37°C on an orbital shaker at 180 rpm for the 5-day-course of protein production. Supernatant was then harvested by centrifugation and purified using one-step Protein A chromatography: after loading, the column was washed with 0.1M Na-phosphate buffer, pH 7.0, elution was achieved with 0.1M glycine, pH 3.5, and protein containing fractions were neutralized immediately by addition of 2M Tris and dialysed into phosphate-buffered saline (PBS) overnight at 4°C. Multimeric fraction was removed using gel filtration with a HiLoad 16/600 Superdex 200 pg column.

### Size exclusion chromatography (SEC)

Shimadzu LC20A Prominence system equipped with a diode array and a refractive index detector was used to perform size exclusion HPLC with Superdex 200 Increase 10/300 GL. The mobile phase buffer was PBS with 200 mM NaCl. Chromatography was performed with a constant flow rate of 0.75 ml/min. A total of 20 μg of protein were loaded on the column for analysis. Gel filtration standard was from Bio-Rad and included proteins of 670, 158, 44, 17 and 1.35 kDa in size.

### Mass spectrometry (MS)

#### Protein analysis

The samples were deglycosylated using PNGase F prior to the measurement. To determine the correct pairing of the heterodimer chains, the proteins were directly injected to a liquid chromatography-electrospray ionization-mass spectrometry (LC-ESI-MS) system (LC: Dionex Ultimate 3000 LC). A gradient from 20 to 80% acetonitrile in 0.05% trifluoroacetic acid (using a Thermo ProSwift™ RP-4H column of 0.2 x 250 mm dimensions) at a flow rate of 8 μL/min was applied (30 min gradient time). Detection was performed with a Q-TOF instrument (Bruker maXis 4G) equipped with the standard ESI source in positive ion, MS mode (range: 750–5000 Da). Instrument calibration was performed using ESI calibration mixture (Agilent). Data was processed using Data Analysis 4.0 (Bruker) and the spectrum was deconvoluted by MaxEnt.

#### Glycan analysis

Glycosylation was analyzed using LC-ESI-MS analysis of peptides originating from protease treatment. The samples were digested in solution. The proteins were S-alkylated with iodoacetamide and digested with Trypsin (Promega).The digested samples were loaded on a BioBasic C18 column (BioBasic-18, 150 x 0.32 mm, 5 μm, Thermo Scientific) using 65 mM ammonium formiate buffer as the aqueous solvent. A gradient from 5% B (B: 100% acetonitrile) to 32% B in 35 min was applied, followed by a 15-min gradient from 32% B to 75% B that facilitates elution of large peptides, at a flow rate of 6 μl/min. Detection was performed with QTOF MS (Bruker maXis 4G) equipped with the standard ESI source in positive ion, DDA mode, switched to MS/MS mode for eluting peaks). MS-scans were recorded (range: 150–2200 Da) and the 3 highest peaks were selected for fragmentation. Instrument calibration was performed using ESI calibration mixture (Agilent).

Manual glycopeptide searches were made using DataAnalysis 4.0 (Bruker). For the quantification of the different glycoforms the peak areas of EICs (Extracted Ion Chromatograms) of the first four isotopic peaks were summed, using the quantification software Quant Analysis (Bruker).

### Differential scanning calorimetry (DSC)

DSC experiments were performed using an MicroCal PEAQ-DSC Automated system (Malvern). Protein samples at 5 μM in PBS were heated from 20°C to 110°C at 1°C/min heating rate, cooled *in situ* and heated again under the same conditions. The second scan was used as a baseline to subtract from the thermogram resulting from the first scan. After normalization, data were evaluated using Origin 7.0 for DSC and fitting was performed using the non-2-state transition mechanism. Melting temperature (Tm) values presented are an average derived from at least three thermal scans.

### Cell surface binding

HER2/neu-positive cell line SK-BR3 and antigen negative cell line MB-MDA468 were cultured in DMEM with 10% fetal calf serum (FCS), 4 mM glutamine and 1x penicillin-streptomycin at 37°C and 5% CO_2_. The cells were harvested and diluted to 100 000 cells/ well, blocked on ice for 30 min with 2% bovine serum albumin (BSA)-PBS, and stained with trastuzumab and trastuzumab-derived constructs in a 3-fold dilution series in blocking buffer on ice for 30 min. The binding of the antibodies was detected after staining with an anti-C_H_2-fluorescein isothiocyanate (FITC) conjugate (Bio-Rad), diluted 1:200 in 2% BSA-PBS for 30 min on ice. To examine the staining with trastuzumab (TRA)-Fab derived constructs, cells were first incubated with a 3-fold dilution series of the antibody fragment starting with 100 nM, and the detection was with anti-kappa-FITC conjugate (Sigma-Aldrich) for TRA-Fab and anti-γ chain-FITC conjugate (Sigma-Aldrich) for TRA-Fab-C_H_3 constructs. Dead cells were gated away using 7-aminoactinomycin D (7-AAD). Mean fluorescence intensity of the live cell population was detected and the values corresponding to the percent of maximal fluorescence were fitted with a 4-parameter curve using Sigma Plot 13.0 program. EC_50_ values presented are derived from two experiments where the individual stainings were performed in duplicates.

### ADCC reporter bioassay

The first test of the biological activity of the antibody was the ADCC Reporter Bioassay (Promega), which gives a readout at an early point in ADCC pathway activation: the activation of gene transcription through the nuclear factor of activated T-cells (NFAT) pathway in the effector cells. The effector cells are engineered Jurkat cells stably expressing the FcγRIIIa receptor, V158 (high affinity) variant, and an NFAT response element driving expression of firefly luciferase. Biological activity of the antibody is quantified through the luciferase produced as a result of NFAT pathway activation and luciferase activity in the effector cell is quantified with luminescence readout.

For this test, 15 000 SK-BR3 cells per well were seeded in 100 μl culture medium and kept at 37°C, 5% CO_2_, in humidified atmosphere overnight. Then the culture medium was removed and replaced with 25 μl RPMI containing 4% low-IgG serum. Antibodies were added in 25 μl in 3-fold serial dilution starting from 20 nM concentration, and finally engineered Jurkat-FcγRIIIa reporter cells were added in 25 μl RPMI with 4% low-IgG serum at a ratio of effector to target cells (E:T) of 5:1. After a 6-h-incubation at 37°C, plates were removed and equilibrated to room temperature. The luminescent substrate was added and the luminescent signal measured in a Tecan microplate reader. The data was fit using 4-parameter curve using Sigma Plot 13.0. EC_50_ values presented are an average of three experiments. HER2/neu-negative MB-MDA468 cell line as target cells were used as a negative control.

### Binding to FcγRIIIa

The relative affinity constant of trastuzumab and the modified constructs towards FcγRIIIa was measured by Biolayer Interferometry (BLI) using an Octet system (Pall Forte Bio Europe) and Forte Bio Acquisition Software in 96-well-microtiter plates at 30°C with orbital sensor agitation of 1000 rpm. FcγRIIIa (R&D Systems) was loaded onto His2 biosensors, a baseline was established in PBS buffer supplemented with 10% (v/v) 10x Kinetics buffer (Pall Forte Bio Europe) followed by 300 s association of the analyte at graded concentrations starting from 2.5 μM. Sensors were then rinsed in 1x Kinetics Buffer for 600 s. Double reference was recorded in 1x Kinetics Buffer and was subtracted from all measurements. Octet Analysis Software version 6.4 was used for automatic data processing. Biosensor data were fit using a 1:1 binding model.

### ADCC assay with NK cells

NK cells were isolated from blood from naïve donors using negative selection. Venous blood was collected into citrate tubes (Beckton Dickinson) and incubated with RosetteSep^TM^ Human NK Cell Enrichment Cocktail (Stemcell Technologies) for 20 min at RT. The mix was overlayed onto a Ficoll-Paque layer in Sep-Mate tubes (Stemcell technologies) and the NK cells were collected in the upper layer after centrifugation at 1200g for 10 min with brake on. After two wash steps with 2% low IgG serum-PBS at 300g for 8 min, cells were counted and their purity was verified by staining with an anti-human CD56 antibody (Stemcell Technologies). They were then added in a E:T ratio of 5:1 to the target SK-BR3 cells or the control MB-MDA468 cells that were seeded on the previous day at 10 000 cells/well into a 96-well plate and preincubated with graded concentrations of test proteins for 20 min at 37°C. Human IgG1 isotype antibody (Sigma) was used at a concentration of 30 nM as a negative control. Cetuximab antibody at a concentration of 66 nM was used to induce lysis of MB-MDA468 cells. Incubation was for 6 h at 37°C. Finally, cell lysis was determined using Cytotox-Glo^TM^ Cytotoxicity Assay (Promega). AAF-Glo^TM^ reagent (Promega) was added, incubated for 15 min at RT and the luminescence was recorded with a Tecan Reader Spectrometer. Percent lysis was calculated from readings of duplicate test wells. Luminescent signals of the wells where the cells were lysed with digitonin according to the manufacturer’s instructions were considered complete lysis and the values corresponding to the cells without antibody coating were considered as background originating from spontaneous lysis and were subtracted.

## Results

### Expression of the domain-exchanged antibody

**Fig 2:**
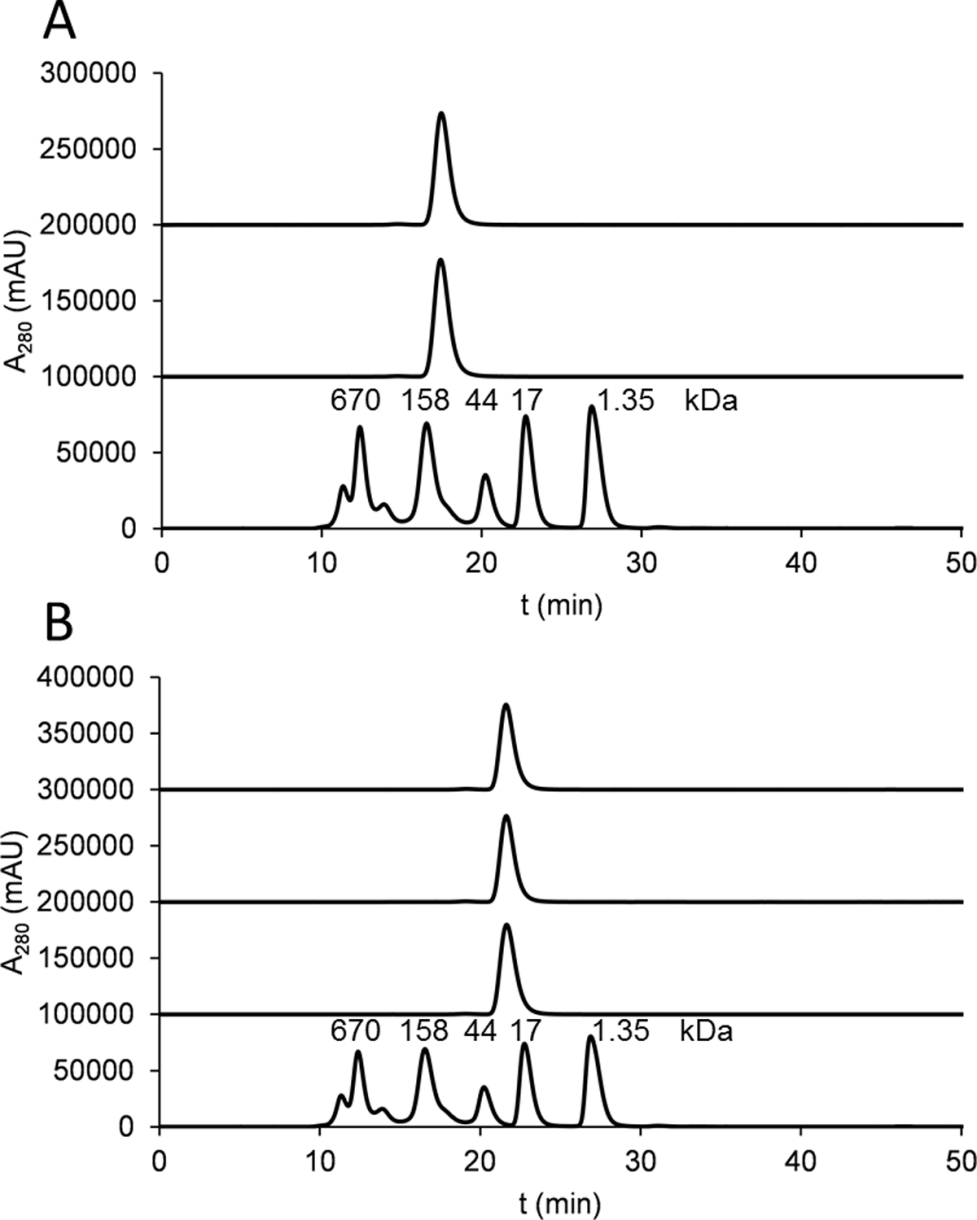
The HPLC profiles of the domain-exchanged antibody and the corresponding Fab-like fragments. (A) From top to bottom: trace for TRA-C_H_3_KiH_, trastuzumab and the gel filtration standard. (B) From top to bottom: trace for TRA-Fab-C_H_3_ZW1,_ TRA-Fab-C_H_3_KiH_, 4D5 Fab and the gel filtration standard.

Chains of the TRA-C_H_3_KiH_ and the corresponding monovalent constructs (schematic representation in Fig 1) were simultaneously transfected into HEK293-6E cells and yields of about 100 mg/l supernatant after Protein A purification were achieved. The solubility of the protein was 1.8 mg/ml after dialysis into PBS, pH 7.4. The protein was over 95% monomeric as shown by HPLC-SEC in native conditions and the multimeric fraction could be removed with a single step of SEC (Fig 2A). With TRA-C_H_3_ZW1_ similar yields and solubility were observed. This mutant was continuously precipitating upon storage and was therefore not used for antigen binding experiments and studies of biological activity.

Mass spectrometry analysis of TRA and TRA-C_H_3_KiH_ showed a homogenous spectrum, in each sample only one variant was detected (S1 Fig). All samples were lacking the terminal lysine on the heavy chain, were completely deglycosylated and all cysteine bridges were formed. For both samples, the measured molecular weight matched the theoretical molecular weight (calculation shown in the S4 Table).

The glycan pattern was investigated with an LC-ESI-MS analysis of peptides originating from protease treatment, and was similar for TRA and TRA-C_H_3_KiH_ (S1 Fig). The major glycoforms are complex type glycans (major glycan GnGnF). Other glycoforms (Man5, GnMF, AGnF, AAF, NaAF, NaNaF and GnGn) were detected as well. In the S5 Table the relative proportions of the different glycoforms are shown (quantified by integration of EIC of the first 4 isotopic peaks). All samples contained less than 1% of the non-glycosylated peptide EEQYNSTYR. The proglycan nomenclature (http://www.proglycan.com/protein-glycosylation-analysis/nomenclature) was used and only one possible isomer is annotated.

The Fab fragment of trastuzumab (TRA-Fab) and the corresponding fragments with exchanged constant domains (TRA-Fab-C_H_3_KiH_ and TRA-Fab-C_H_3_ZW1_) were expressed in HEK293-6E cells and purified with Protein A affinity chromatography at a similar yield to the unmodified Fab fragment of about 100 mg/l. The amount of aggregate was 10% for TRA-Fab-C_H_3_KiH_ and 9% TRA-Fab-C_H_3_ZW1_ and could be removed with a single step of SEC (Fig 2B).

### Thermal stability

**Fig 3:**
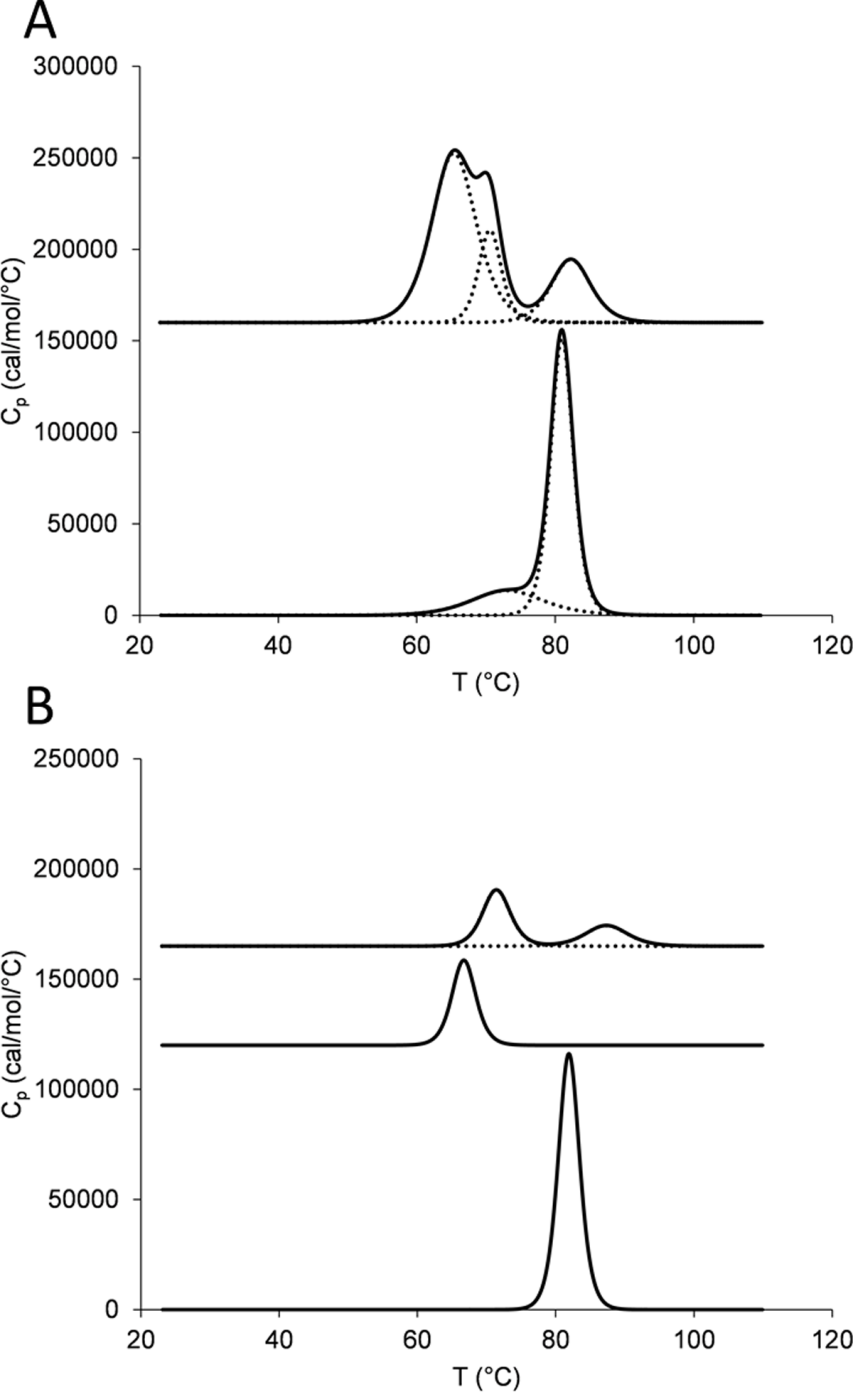
Thermal stability of the domain-exchanged antibody and the Fab-like fragments. (A) Deconvolution of the thermogram for TRA-C_H_3_KiH_ (upper trace) and trastuzumab (lower trace), (B) from top to bottom: deconvolution of the thermogram of TRA-Fab-C_H_3_ZW1,_ TRA-Fab-C_H_3_KiH_ and 4D5 Fab.

For trastuzumab, differential scanning calorimetry revealed two transitions: at 72.60±0.26 and 80.69±0.20°C (Fig 3A), which fits well to published data [19]. For the TRA-C_H_3 three transitions could be observed: at 65.25±0.09°C, 70.30±0.07°C and 81.95±0.01°C. The last transition corresponds to the melting of the unmodified C_H_3 domain and the first two transitions result hence from several sequential phenomena such as denaturation of the C_H_3_KiH_ domains, denaturation of the C_H_2 domains, dissociation of C_H_2 and C_H_3 domains, thermal denaturation of the variable domains of the Fab fragment and their dissociation from the newly introduced pair of C_H_3 domains [20–22]. A similar degree of thermal destabilization upon constant domain exchange was observed for monovalent fragment TRA-Fab-C_H_3_KiH_, which denatured in a single thermal transition at 66.58±0.10°C while for unmodified TRA-Fab a Tm of 81.87±0.13°C could be observed (Fig 3B). Destabilization to melting temperatures of 69°C or less has been reported previously for heterodimers of C_H_3 domains with mutations introduced at the core interface [23]. TRA-Fab-C_H_3_ZW1_ was of a higher thermal stability with 2 transitions at 71.08±0.25°C and 86.87±0.54°C, resulting from design of heterodimeric C_H_3 with improved stability [16] and an additional C-terminal cysteine bond [17].

### Optimization of the variable domain—C_H_3 interface

To improve the interaction contacts between variable domains and newly introduced constant domains, several point mutations and their combinations were introduced in the sequence of the original TRA-C_H_3_KiH_ construct (S2 Fig, S2 Table). A structural model of the TRA-C_H_3_KiH_ Fab part was constructed to identify possible mutation sites in the novel domain interface. Potential mutations that could contribute to optimization of the novel variable and constant domain interface were identified by visual inspection of the modelled molecule and comparison to the variable domain–C_H_1-C_L_ interface. A summary of the rationales of the selected mutations and of the effects on thermostability is given in S2 Table. HPLC-SEC of all constructs was performed and the aggregate amounted to less than 5% of total protein for all constructs tested (S3 Fig). All constructs eluted at a time typical for trastuzumab, corresponding to 158 kDa as indicated by the molecular weight marker. Thermostability of the mutants in comparison with TRA-C_H_3_KiH_ is presented in S4 Fig.

### Heavy chain

#### Positions Ser375 and Asp376

The alteration of the amino acid at position 376 aimed at an increase in hydrophobic packing of the interface that could exert a stabilizing effect on the conformational rigidity, and was first attempted by the introduction of a Leu residue. However, this both destabilized the molecule (Tm at 63.90±0.01, 70.14±0.00 and 82.45±0.01°C) and decreased the solubility to 630 μg/ml, which could be explained by greater solvent exposure of the Leu residue as suggested by the structural model. The destabilizing effect could however be alleviated by the mutation Ser375Asp. Double mutant Ser375Asp/Asp376Leu exhibited both an increased thermal stability (Tm at 64.40±0.12, 69.76±0.19 and 82.60±0.39°C) and an improved solubility over 2.6 mg/ml in PBS. To account either for overpacking of the hydrophobic cavity or to introduce a residue that is of a more appropriate van der Waals’ radius, the smaller residue Val was introduced at the position 376, and the Asp at the position 375 remained as a residue that could form hydrogen bonding to the water network between the variable and constant domains.

#### Position Phe404

The mutation Phe404Tyr was introduced to decrease the hydrophobicity of the formerly buried interface residue which is possibly solvent exposed in the variable-domain–C_H_3 interface. Moreover, the corresponding residue in the C_H_1 domain identified by the structural alignment is also Tyr (Tyr180). The mutant was created to increase solubility of the domain-exchanged antibody. This mutant exhibited both an increase in thermostability (Tm at 66.56±0.18, 70.67±0.29 and 82.81±0.01°C) as well as an improvement in the solubility in PBS that was now 4.4 mg/ml. As the analysis of the structural model suggested that Asp375 could also form a hydrogen bond to the Tyr404, a combination of the three mutations Ser375Asp/Asp376Val/Phe404Tyr was introduced into one chain. Judging from the thermogram overlay (S4 Fig), the combined mutant was of higher thermostability than the parental molecule TRA-C_H_3_KiH_ H:Phe404/Tyr.

#### Position Gly10 in the variable heavy domain

Upon the visual inspection of the heavy chain structure, the stretch of amino acids Gly8/Gly9/Gly10 appeared a promising target for mutagenesis, especially as at the position 10 a bulkier amino acid characteristic for the motive Gly8/Ala9/Glu10 is common in several antibody germlines of human variable heavy domain families V_H_1, V_H_5 and V_H_7 [24]. In the proposed mutant Gly10Arg a stabilizing salt bridge could be formed to Glu430 of the newly introduced C_H_3 domain. The mutant Gly10Arg indeed exhibited an improved stability in comparison with the unmodified TRA-C_H_3_KiH_ judging from overlays of their thermograms (S4 Fig).

### Light chain

#### Position Ser375

At this position a mutation to Arg was intended to introduce a stabilizing salt bridge to the residue Glu105 of the variable domain of the light chain, similarly as in the wild-type Fab sequence. This mutant indeed exhibited an improved thermostability. The mutant Ser375Lys was constructed to test another basic amino acid in this position, however the thermostability of this construct was on the level of the unmodified TRA-C_H_3_KiH_ judging from the thermogram overlays in S4 Fig.

#### Position Phe404

The mutation Phe404Tyr was introduced because Tyr possibly could form a stabilizing hydrogen bond with Glu105 and the corresponding residue in the C_κ_ domain identified by the structural alignment was also a Tyr (Tyr173). The mutation indeed increased the thermostability of the modified protein as intended (Tm at 66.01±0.06, 70.63±0.13 and 82.77±0.07°C).

#### Position Glu430

The mutation Glu430Gln was introduced to remove unbalanced charge as this residue in the unmodified Fc fragment forms a salt bridge with Lys338 located in the C_H_2 domain. A positive effect on thermal stability of this mutant could be established (Tm at 66.01±0.36, 70.49±0.19 and 82.87±0.18°C).

#### Interface mutations of the light chain do not stabilize in combination

The mutant with the combination of Ser375Arg and Phe404Tyr was not more stable than TRA-C_H_3_KiH_ L:Phe404Tyr alone (Tm at 64.78±0.30, 70.30±0.24 and 82.72±0.10°C). A possible explanation for this outcome is that both mutated residues were selected because of possible interactions with residue Glu105 of the variable domain, but the single favorable interactions are not additive or do not occur simultaneously. The combination of stabilizing mutations Ser375Arg and Glu430Gln resulted in a molecule less stable than any of the stabilized mutants alone (Tm at 64.11±0.58, 69.28±0.31 and 82.81±0.15°C) and even less stable than the unmodified TRA-C_H_3_KiH_. Even a less stable molecule was produced by combining the mutations Phe404Tyr and Glu430Gln, where the melting points of the first and the second thermal transition were decreased from those characteristic for unmodified TRA-C_H_3_KiH_ by 5.9 and 4.1°C respectively. The protein with combined mutations TRA-C_H_3_KiH_ L:Ser375Arg/Phe404Tyr/Glu430Gln was still less stable than the unmodified TRA-C_H_3_KiH_ with Tms of the first and the second thermal transition decreased by 1.3 and 0.7°C respectively and less evolved heat upon denaturation (S4 Fig). Possible explanations for these outcomes are structural rearrangements upon mutation which cannot be predicted by the used modeling approach.

#### Combination of stabilizing mutations in the heavy and the light chain of the TRA-C_H_3_KiH_

The most potent stabilizing mutations, Ser375Asp/Asp376Val/Phe404Tyr in the heavy chain and Phe404Tyr in the light chain, were combined to yield the mutant H: Ser375Asp/Asp376Val/Phe404Tyr//L:Phe404Tyr that was superior to unmodified TRA-C_H_3_KiH_ in thermostability, but not better than TRA-C_H_3_KiH_ L:Phe404Tyr (Tm at 65.89±0.17, 70.643±0.27 and 82.69±0.26°C). The stabilizing effect was potentiated by the combination of the single point mutation Phe404Tyr in the light chain and in the heavy chain (Tm at 66.57±0.20, 71.06±0.19 and 82.71±0.23°C) and this could also be inferred from the overlay of the thermograms in S4C Fig. The expression level of this protein was about 200 mg/l supernatant and the solubility in PBS was 5.5 mg/ml. In the MS analysis of this protein only one heterodimeric species could be detected (S1 Fig) and its molecular weight matched the calculated molecular weight of the heterodimer (S4 Table), similarly as found for trastuzumab and TRA-C_H_3_KiH_. The glycosylation pattern was identical to trastuzumab and TRA-C_H_3_KiH_ (S1 Fig).

### Cell surface binding

**Fig 4:**
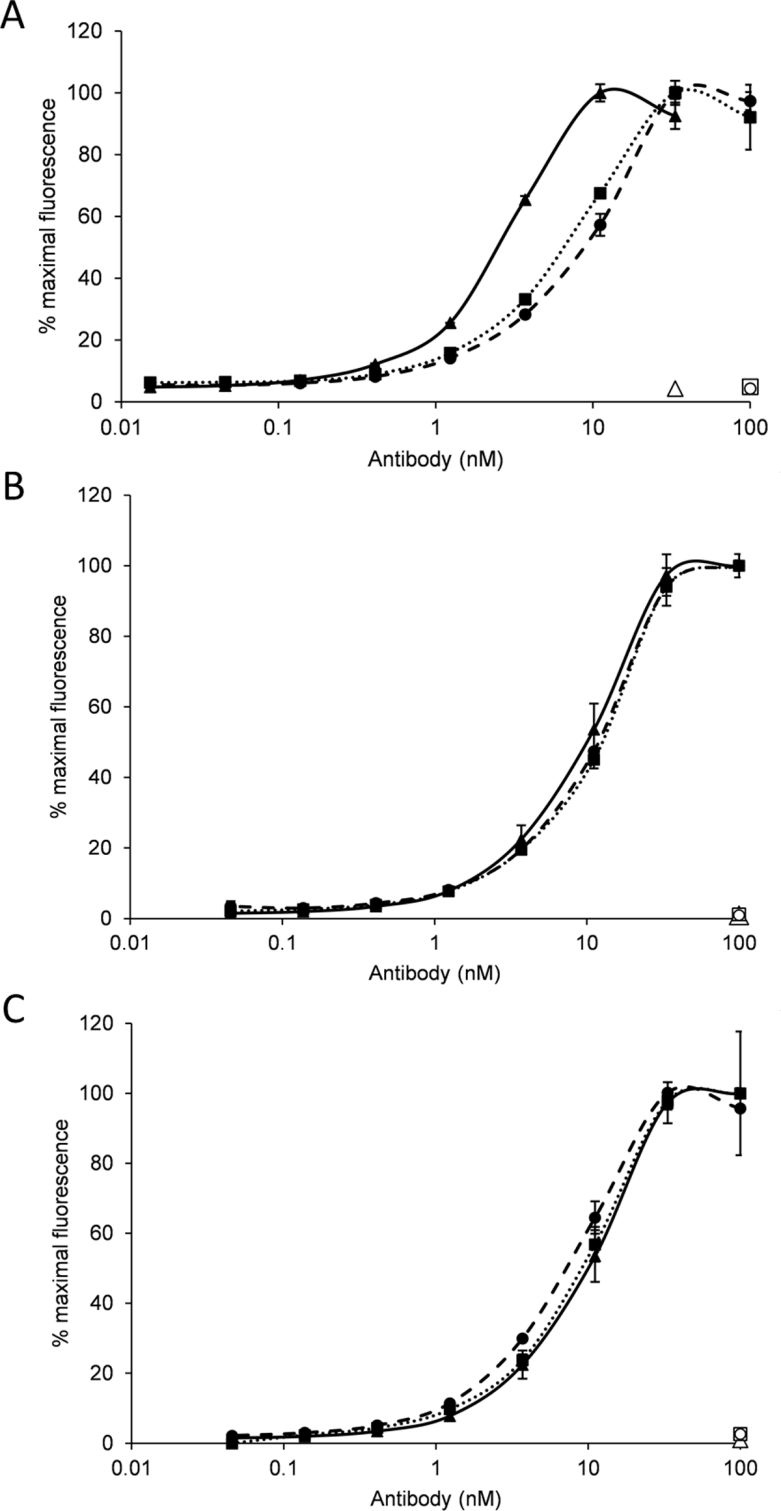
Cell surface binding of domain-exchanged antibody and the Fab-like fragments to SK-BR3 cells. (A) Binding to HER2/neu positive SK-BR3 cells of trastuzumab (full line—full triangles), TRA-C_H_3_KiH_ (dotted line—full squares) and TRA-C_H_3_KiH_ H:Phe404Tyr//L:Phe404Tyr (dashed line—full circles). No binding to HER2/neu negative MB-MDA468 could be detected with either trastuzumab (empty triangle), TRA-C_H_3_KiH_ (empty circle) or TRA-C_H_3_KiH_ H:Phe404Tyr//L:Phe404Tyr (empty square). (B) Binding to HER2/neu positive SK-BR3 cells of trastuzumab Fab (full line—full triangles), TRA-Fab-C_H_3_KiH_ (dotted line—full squares) and TRA-Fab-C_H_3_KiH_ H:Phe404Tyr//L:Phe404Tyr (dashed line—full circles), (C) Binding to HER2/neu positive SK-BR3 cells of trastuzumab Fab (full line—full triangles), TRA-Fab-C_H_3_ZW1_ (dotted line—full squares) and TRA-Fab-C_H_3_ZW1_ H:Phe404Tyr//L:Phe404Tyr (dashed line—full circles). No binding to HER2/neu negative MB-MDA468 could be detected with any of the constructs (corresponding empty markers in each panel).

The full length and the Fab-like domain-exchanged antibody variants were tested for binding to the cell surface of the strongly HER2/neu-positive cell line SK-BR3 (Fig 4). After staining with graded concentrations of trastuzumab, TRA-C_H_3_KiH_ antibody or its interface optimized variant H:Phe404Tyr//L:Phe404Tyr, the binding of the antibodies to the cell surface was determined by measuring the mean fluorescence intensity of the bound detection reagent, the anti-C_H_2 antibody whose binding epitope maps to the C-terminal part of the C_H_2 domain [25]. TRA-C_H_3_KiH_ H:Phe404Tyr//L:Phe404Tyr bound to the SK-BR3 cells with an EC_50_ of 9.2±1.4 nM and the non-optimized version with an EC_50_ of 9.4±0.8 nM. Both were weaker than trastuzumab, for which an EC_50_ of 2.2±0.4 nM was measured. The constructs showed no staining of the HER2/neu—negative cell line MB-MDA468.

The lower binding affinity could not be ascribed to a diminished monovalent binding as the EC_50_ values for cell surface staining of Fab-like fragments with exchanged constant domains were very similar to that measured for TRA-Fab: 11.8±0.8 nM for TRA-Fab-C_H_3_KiH_ and 8.5±1.2 nM for TRA-Fab-C_H_3_ZW1_ compared well with 9.8±0.5 nM determined for the unmodified TRA-Fab (Fig 4B and 4C). Similar EC_50_ could be observed also for TRA-Fab-C_H_3_KiH_ H:Phe404Tyr//L:Phe404Tyr (11.1±1.1 nM) and TRA-Fab-C_H_3_ZW1_ H:Phe404Tyr//L:Phe404Tyr (11.6±1.4 nM).

### Domain-exchanged antibody elicits a potent activation of NFAT response and an ADCC similar to the parental antibody

**Fig 5:**
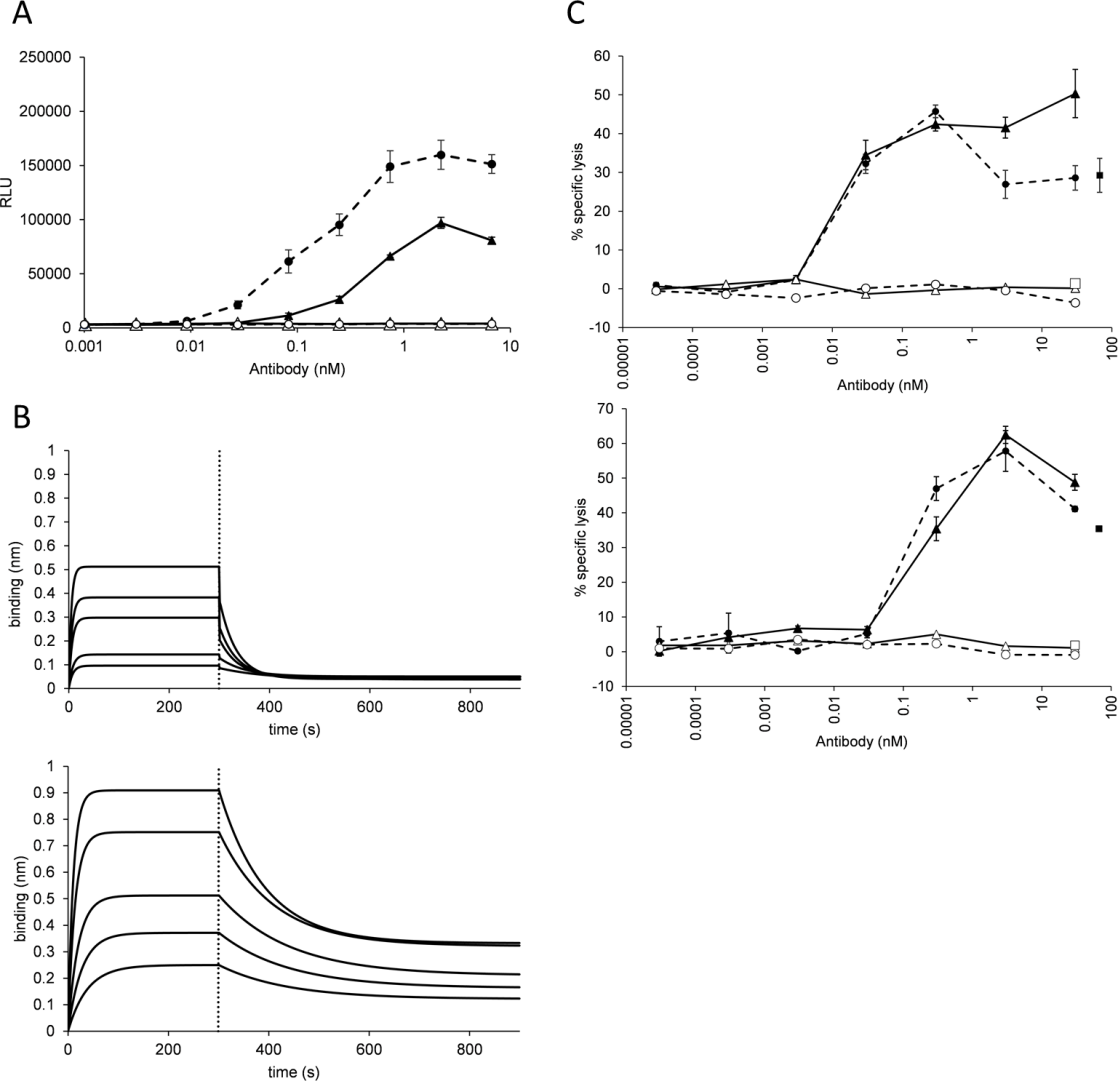
Domain-exchanged antibody elicits a more potent activation of NFAT pathway in FcγRIIIa-overexpressing T-cells than the parental antibody trastuzumab due to stronger binding to FcγRIIIa. (A) T-cell activation was tested at an E:T ratio of 5:1 (upper pannel) and 15:1 (lower panel). The activation of T-cells in response to HER2/neu positive SK-BR3 cells coated with trastuzumab (full line–full triangles) or TRA-C_H_3_KiH_ H:Phe404Tyr//L:Phe404Tyr (dashed line—full circles) and HER2/neu—negative MB-MDA468 cells incubated with trastuzumab (full line–empty triangles) or TRA-C_H_3_KiH_ H:Phe404Tyr//L:Phe404Tyr (dashed line—empty circles). (B) Results of BLI assay for trastuzumab (upper panel) and TRA-C_H_3_KiH_ H:Phe404Tyr//L:Phe404Tyr (lower panel) binding to FcγRIIIa. The curves correspond to the response of 2500, 1250, 625, 312.5 and 156.3 nM antibody. Dotted lines indicate the start of dissociation. (C) ADCC assay with NK cells from two donors (upper and lower panel) with trastuzumab (full line–full triangles) or TRA-C_H_3_KiH_ H:Phe404Tyr//L:Phe404Tyr (dashed line—full circles) and HER2/neu—negative MB-MDA468 cells incubated with trastuzumab (full line–empty triangles) or TRA-C_H_3_KiH_ H:Phe404Tyr//L:Phe404Tyr (dashed line—empty circles). Incubation of target cells with isotype control is indicated with an empty square and the lysis of the control MB-MDA468 cells, induced with Cetuximab, with a full square.

TRA-C_H_3_KiH_ H:Phe404Tyr//L:Phe404Tyr was tested in an ADCC reporter bioassay that measures the activation of NFAT pathway of FcγRIIIa-overexpressing T-cells. The EC_50_ of activation was 0.22±0.06 nM in comparison with 0.47±0.04 nM of trastuzumab at an E:T ratio of 5:1 (Fig 5A). Additionally, the engineered T-cell response was higher with the domain-exchanged construct. No response could be measured when they were tested with antibodies applied with the antigen-negative cell line MB-MDA468. To be able to compare the assay results with the manufacturer’s data, we have performed the experiment with trastuzumab at an E:T ratio of 15:1 and could determine the EC_50_ of 0.094 nM, comparable with 0.067 nM as suggested by the product manual and published data [26]. The binding to the isolated FcRγIIIa was then evaluated with the BLI system and a binding constant of 1.0 x 10^−7^ M for TRA-C_H_3_KiH_ H:Phe404Tyr//L:Phe404Tyr in comparison with 2.69 x 10^−7^ M for trastuzumab could be determined (Fig 5B). The binding affinity of trastuzumab to FcγRIIIa was similar as described before (252 nM) [27].

The domain-exchanged antibody was able to elicit NK-cell mediated ADCC to specifically lyse target SK-BR3 cells to an extent similar to the parental antibody (Fig 5C). There was no effect on the target-negative Her-1 positive cell line MB-MDA468, which could however be lysed with the Her-1 targeting antibody Cetuximab. At the same time, there was no specific cell lysis when the target cells were incubated only with effector cells or with isotype antibody and the effector cells. With one donor, the EC_50_ for both trastuzumab and the domain-exchanged antibody was between 0.1 and 0.01 nM, which agrees well with the literature cited value of 0.02 nM [4]. With the NK cells from the second donor, the EC_50_ was between 1 and 0.1 nM for both tested compounds.

## Discussion and conclusions

We set out to construct an antibody-like molecule with a C_H_3-homodimer replacing the C_H_1- C_κ_ domain pair in the Fab fragment. Containing only natural amino acid sequences of the domains that have been brought into proximity, a heterodimerization motif to allow selective pairing of variable domains and a C-terminal cysteine bond connecting the newly introduced C_H_3 dimer, the domain-exchanged antibody could be produced at a high level in a standard lab-scale mammalian expression system. With a series of point mutations aimed at stabilization of the interface interactions of the variable and C_H_3 domains we obtained information on the possibility of improving the biophysical properties of the construct. While the structural model of the TRA-C_H_3_KiH_ Fab helped to identify single stabilizing mutations, the combination of different mutations was often not having an additional stabilizing effect, sometimes even deteriorating the former stabilizing effects. This can probably be attributed to more complex structural movements or rearrangements not captured by the relatively simple and static structural model. More extensive conformational searching or sampling procedures might allow for the coverage of such effects. Regarding the relatively small number of amino acid exchanges that have been introduced, it is likely that its solubility can be further improved by performing a more extensive mutagenesis.

The surface staining of the HER2/neu positive cell line SK-BR3 with the domain-exchanged antibody was found to be less strong than with trastuzumab. Cell surface staining implies the engagement of both binding sites of the bivalent antibody, which could amplify a negative effect of constant domain exchange on antigen binding affinity mediated by the variable domains. Initially, we ascribed this property to imperfect heterodimerization typical of the minimal “Knobs-into-Holes” mutagenesis applied [15]. This could be improved with more efficient heterodimerization strategies, such as combination with electrostatic steering [28, 29], interface modification as described for the novel BEAT antibodies [30] or the very stable ZW1 motive [16]. At the same time, MS analysis of the domain-exchanged antibody suggested a very high proportion of correctly heterodimerized species. Cell surface staining with monovalent Fab-like constant domain-exchanged fragments was very similar to the trastuzumab Fab. For these fragments there is only one binding site for Protein A mapping onto V_H_ domain, theoretically leading to heterodimeric product contaminated only with the V_H_-C_H_3 –“Knob” homodimeric species after the purification step. In this view, purification-assisted techniques could assist in increasing the yield of heterodimeric whole-length antibody, but would involve tagging or mutagenizing of one of the chains to allow selective affinity purification.

In spite of the weaker cell binding, TRA-C_H_3_KiH_ H:Phe404Tyr//L:Phe404Tyr mediated a more potent activation of NFAT pathway in FcγRIIIa-overexpressing effector cells than observed for trastuzumab, which could be explained by the stronger binding to FcγRIIIa. This phenomenon was interesting and unexpected, especially considering the weaker target cell surface binding. As the N-glycan pattern was similar for trastuzumab and the domain-exchanged antibodies, such changes could most likely be attributed to the conformational change in the hinge region of the antibody caused by the exchange of the constant domains of the Fab. Fcγ receptor binds asymmetrically in the region of the hinge between the C_H_2 domains [31] and the receptor binding spreads the C_H_2 domains apart [32, 33]. The functional data revealing the differences in FcγR binding describes mainly the effects of the mutations in the Fc-hinge region [34, 35], but also mutational variants located at the C_H_2-C_H_3 interface and even mutagenesis of C_H_3 domains [30, 36], signifying that also the relatively remote residues influence the conformation of the hinge region, presumably through the adjustment of the position or restriction of the movement of the C_H_2 domains [13]. The ability of the domain-exchanged antibody to incite a specific ADCC mediated by NK cells was similar to the effect of the parental antibody in spite of the weaker binding to the surface of target cells.

It is widely accepted that sustained circulation levels of IgG1 are largely dependent on their interaction with neonatal Fc receptor (FcRn) [37]. A majority of the residues involved in this interaction are located in the junction of the C_H_2 and C_H_3 domains, however several residues located in the C_H_3 domain govern importantly this interaction including His433 and His435 that conduct the pH-dependency of the binding [38]. Early reports on scFv fragments fused to C_H_3 domains with connecting peptides describe an improved tumor targeting functionality of such agents as compared to their monomers due to a longer biological half-life resulting from a higher molecular weight and gain in avidity, but also further improvements in serum persistence of the format with an increased solvent accessibility of residues His433 and Asn434 of the C_H_3 domain [39]. It will be very interesting to study this interaction with the domain-exchanged antibody that harbors an additional site that could potentially engage FcRn in pH-dependent manner as well as the influence of the domain exchange on the pharmacokinetic properties of the antibody modified in this way *in vivo*.

With the increasing interest in the field of bispecific antibodies several strategies have been developed to overcome the promiscuous pairing of light chains with heterodimerized heavy chains, such as use of an scFv and an Fab fragment on each heavy chain, “CrossMab” format [40] or application of common light chain [41, 42]. As one option, a C_H_3 domain-exchanged Fab fragment at one arm could be combined with an unmodified Fab of a different specificity at the other arm of a heterodimeric antibody.

Finally, it would be attractive to explore the possibility of introducing novel antigen binding sites into the context of the C_H_3 domains at an unusual position, analogously as described for Fc fragments with antigen binding capability (Fcabs) [4], which incorporated into a complete antibody lead to a bispecific molecule (mAb^2^). Such molecule with antigen-binding C_H_3 domains would have the potential of harboring even an additional antigen specificity or ability of multivalent binding. Positioning of the antigen-binding C_H_3 domains at the C-terminal structural loops of the constant domain of the modified Fab fragment allows a small distance between the binding sites for the novel antigen and the effector molecules. This spatial proximity could cause a potentiated cellular response, as described for other immune synapse bridging molecules that allow a close contact of effector and target cells [43].

To conclude, the principles used in the construction of domain-exchanged antibody described here could be implemented for construction of several novel bispecific and multispecific binding agents.

## Supporting information

S1 Fig Mass spectrometry analysis of the domain-exchanged antibody. (A) intact mass analysis of TRA, TRA-C_H_3_KiH and_ TRA-C_H_3_KiH_ H:Phe404Tyr//L:Phe404Tyr_,_ (B) glycan pattern analysis of trastuzumab and the domain exchanged antibody variants. The peak heights in the MS spectra roughly reflect the molar ratios of the glycoforms (note that more than one charge state is present per glycoform).
(TIF)

S2 Fig Interface mutations to improve the stability and solubility of the domain-exchanged antibody. (A) interface mutations in the heavy chain, (B) interface mutations in the light chain, (C) surfaced cartoon diagram of TRA-CH3KiH H:Phe404Tyr//L:Phe404Tyr. V_H_: blue, V_κ_: green, C_H_3: gray, mutated residues: teal.
(TIF)

S3 Fig HPLC profiles of domain-exchanged antibody interface mutants. (A) interface mutants modified in the heavy chain, (B) interface mutants modified in the light chain, (C) interface mutants modified in both chains.
(TIF)

S4 Fig Thermostability of domain-exchanged antibody interface mutants. (A) thermograms of interface mutants modified in the heavy chain, (B) thermograms of interface mutants modified in the light chain, (C) thermograms of the interface mutants modified in both chains. Thermogram of the parental construct TRA-CH3KiH is depicted with dashed line and thermograms of the mutants with full line. Where the comparison to other mutants is shown, the thermogram of TRA-CH3KiH L:Phe404Tyr is depicted with a dotted line and the thermogram of TRA-CH3KiH H:Phe404Tyr with a dash-dot-dash line.
(TIF)

S1 Table Amino acid sequences of the domain-exchanged antibody and the Fab-like constructs. The lettering is in the color code corresponding to the construct schematic in Fig 1. Mutations driving the heterodimerization are in red.
(DOCX)

S2 Table List of point mutations and their combinations introduced to optimize the novel interface between variable Fab and C_H_3 domains. Melting points of the mutants as determined with DSC are given in bold and are average of at least three experiments.
(DOCX)

S3 Table List of oligonucleotides used to introduce interface point mutations and their combinations into the TRA-C_H_3 constructs. (DOCX)

S4 Table Theoretical molecular weight of TRA, TRA-C_H_3_KiH_ and TRA-C_H_3_KiH_ H:Phe404Tyr//L:Phe404Tyr. The theoretical mass is calculated by the summation of all chains (2*HC + 2*LC), minus the number of cysteine bridges multiplied by two (-32, in the theoretical mass cysteines are reduced–so the hydrogen mass has to be subtracted), plus the number of glycosylation sites (+2, deglycosylation will lead to N ➔ D conversion) and +1 for the charge. The theoretical masses were calculated using the online tool peptide mass (http://web.expasy.org/peptide_mass/**).**
(DOCX)

S5 Table The relative proportions of the different glycoforms of TRA, TRA-C_H_3_KiH_ and TRA-C_H_3_KiH_ H:Phe404Tyr//L:Phe404Tyr. Proportion of glycoforms in % including the non-glycosylated peptide. The proglycan nomenclature (http://www.proglycan.com/protein-glycosylation-analysis/nomenclature) was used and only one possible isomer is annotated.
(DOCX)

## References

[1] Bork P, Holm L, Sander C. The immunoglobulin fold. Structural classification, sequence patterns and common core. J Mol Biol. 1994 9 30; 242(4): 309–20. doi: 10.1006/jmbi.1994.1582 7932691 10.1006/jmbi.1994.1582

[2] Murzin AG, Brenner SE, Hubbard T, Chothia C. SCOP: a structural classification of proteins database for the investigation of sequences and structures. J Mol Biol. 1995 4 7; 247(4): 536–40. doi: 10.1006/jmbi.1995.0159 7723011 10.1006/jmbi.1995.0159

[3] Dimitrov DS. Engineered CH2 domains (nanoantibodies). MAbs. 2009 Jan-Feb; 1(1): 26–8. 20046570 10.4161/mabs.1.1.7480 PMC2715188

[4] Wozniak-Knopp G, Bartl S, Bauer A, Mostageer M, Woisetschlager M, Antes B, et al Introducing antigen-binding sites in structural loops of immunoglobulin constant domains: Fc fragments with engineered HER2/neu-binding sites and antibody properties. Protein Eng Des Sel. 2010 4; 23(4): 289–97. doi: 10.1093/protein/gzq005 20150180 10.1093/protein/gzq005

[5] Leung KM, Batey S, Rowlands R, Isaac SJ, Jones P, Drewett V, et al A HER2-specific Modified Fc Fragment (Fcab) Induces Antitumor Effects Through Degradation of HER2 and Apoptosis. Mol Ther. 2015 11; 23(11): 1722–33. doi: 10.1038/mt.2015.127 26234505 10.1038/mt.2015.127 PMC4817942

[6] Gong R, Vu BK, Feng Y, Prieto DA, Dyba MA, Walsh JD, et al Engineered human antibody constant domains with increased stability. J Biol Chem. 2009 5 22; 284(21): 14203–10. doi: 10.1074/jbc.M900769200 19307178 10.1074/jbc.M900769200 PMC2682868

[7] Wozniak-Knopp G, Stadlmann J, Ruker F. Stabilisation of the Fc fragment of human IgG1 by engineered intradomain disulfide bonds. PLoS One. 2012; 7(1): e30083 doi: 10.1371/journal.pone.0030083 22272277 10.1371/journal.pone.0030083 PMC3260182

[8] Traxlmayr MW, Faissner M, Stadlmayr G, Hasenhindl C, Antes B, Ruker F, et al Directed evolution of stabilized IgG1-Fc scaffolds by application of strong heat shock to libraries displayed on yeast. Biochim Biophys Acta. 2012 4; 1824(4): 542–9. doi: 10.1016/j.bbapap.2012.01.006 22285845 10.1016/j.bbapap.2012.01.006 PMC3787792

[9] Rothlisberger D, Honegger A, Pluckthun A. Domain interactions in the Fab fragment: a comparative evaluation of the single-chain Fv and Fab format engineered with variable domains of different stability. J Mol Biol. 2005 4 8; 347(4): 773–89. doi: 10.1016/j.jmb.2005.01.053 15769469 10.1016/j.jmb.2005.01.053

[10] Teerinen T, Valjakka J, Rouvinen J, Takkinen K. Structure-based stability engineering of the mouse IgG1 Fab fragment by modifying constant domains. J Mol Biol. 2006 8 25; 361(4): 687–97. doi: 10.1016/j.jmb.2006.06.073 16876195 10.1016/j.jmb.2006.06.073

[11] Lesk AM, Chothia C. Elbow motion in the immunoglobulins involves a molecular ball-and-socket joint. Nature. 1988 9 8; 335(6186): 188–90. doi: 10.1038/335188a0 3412476 10.1038/335188a0

[12] Matsumiya S, Yamaguchi Y, Saito J, Nagano M, Sasakawa H, Otaki S, et al Structural comparison of fucosylated and nonfucosylated Fc fragments of human immunoglobulin G1. J Mol Biol. 2007 5 4; 368(3): 767–79. doi: 10.1016/j.jmb.2007.02.034 17368483 10.1016/j.jmb.2007.02.034

[13] Teplyakov A, Zhao Y, Malia TJ, Obmolova G, Gilliland GL. IgG2 Fc structure and the dynamic features of the IgG CH2-CH3 interface. Mol Immunol. 2013 11; 56(1–2): 131–9. doi: 10.1016/j.molimm.2013.03.018 23628091 10.1016/j.molimm.2013.03.018

[14] Shen Y, Zeng L, Zhu A, Blanc T, Patel D, Pennello A, et al Removal of a C-terminal serine residue proximal to the inter-chain disulfide bond of a human IgG1 lambda light chain mediates enhanced antibody stability and antibody dependent cell-mediated cytotoxicity. MAbs. 2013 May-Jun; 5(3): 418–31. doi: 10.4161/mabs.24291 23567210 10.4161/mabs.24291 PMC4169035

[15] Ridgway JB, Presta LG, Carter P. 'Knobs-into-holes' engineering of antibody CH3 domains for heavy chain heterodimerization. Protein Eng. 1996 7; 9(7): 617–21. 8844834 10.1093/protein/9.7.617

[16] Von Kreudenstein TS, Escobar-Carbrera E, Lario PI, D'Angelo I, Brault K, Kelly J, et al Improving biophysical properties of a bispecific antibody scaffold to aid developability: quality by molecular design. MAbs. 2013 Sep-Oct; 5(5): 646–54. doi: 10.4161/mabs.25632 23924797 10.4161/mabs.25632 PMC3851217

[17] Wozniak-Knopp G, Ruker F. A C-terminal interdomain disulfide bond significantly stabilizes the Fc fragment of IgG. Arch Biochem Biophys. 2012 10 15; 526(2): 181–7. doi: 10.1016/j.abb.2012.03.024 22483683 10.1016/j.abb.2012.03.024

[18] Schymkowitz J, Borg J, Stricher F, Nys R, Rousseau F, Serrano L. The FoldX web server: an online force field. Nucleic Acids Res. 2005 7 1; 33(Web Server issue): W382–8. doi: 10.1093/nar/gki387 15980494 10.1093/nar/gki387 PMC1160148

[19] Ionescu RM, Vlasak J, Price C, Kirchmeier M. Contribution of variable domains to the stability of humanized IgG1 monoclonal antibodies. J Pharm Sci. 2008 4; 97(4): 1414–26. doi: 10.1002/jps.21104 17721938 10.1002/jps.21104

[20] Tischenko VM, Zav'yalov VP, Medgyesi GA, Potekhin SA, Privalov PL. A thermodynamic study of cooperative structures in rabbit immunoglobulin G. Eur J Biochem. 1982 9 1; 126(3): 517–21. 7140745 10.1111/j.1432-1033.1982.tb06811.x

[21] Tischenko VM, Abramov VM, Zav'yalov VP. Investigation of the cooperative structure of Fc fragments from myeloma immunoglobulin G. Biochemistry. 1998 4 21; 37(16): 5576–81. doi: 10.1021/bi972647a 9548942 10.1021/bi972647a

[22] Vermeer AW, Norde W. The thermal stability of immunoglobulin: unfolding and aggregation of a multi-domain protein. Biophys J. 2000 1; 78(1): 394–404. doi: 10.1016/S0006-3495(00)76602-1 10620303 10.1016/S0006-3495(00)76602-1 PMC1300647

[23] Dall'Acqua W, Simon AL, Mulkerrin MG, Carter P. Contribution of domain interface residues to the stability of antibody CH3 domain homodimers. Biochemistry. 1998 6 30; 37(26): 9266–73. doi: 10.1021/bi980270i 9649307 10.1021/bi980270i

[24] Lefranc MP. IMGT, the international ImMunoGeneTics information system [database: Internet]; 2009 [cited 2017/05/22]. Available from: http://www.imgt.org 10.1093/nar/gkn838 PMC2686541 18978023

[25] Traxlmayr MW, Hasenhindl C, Hackl M, Stadlmayr G, Rybka JD, Borth N, et al Construction of a stability landscape of the CH3 domain of human IgG1 by combining directed evolution with high throughput sequencing. J Mol Biol. 2012 10 26; 423(3): 397–412. doi: 10.1016/j.jmb.2012.07.017 22846908 10.1016/j.jmb.2012.07.017 PMC3469823

[26] Cheng ZJ, Garvin D, Paguio A, Moravec R, Engel L, Fan F, et al Development of a robust reporter-based ADCC assay with frozen, thaw-and-use cells to measure Fc effector function of therapeutic antibodies. J Immunol Methods. 2014 12 01; 414: 69–81. doi: 10.1016/j.jim.2014.07.010 25086226 10.1016/j.jim.2014.07.010

[27] Lazar GA, Dang W, Karki S, Vafa O, Peng JS, Hyun L, et al Engineered antibody Fc variants with enhanced effector function. Proc Natl Acad Sci U S A. 2006 3 14; 103(11): 4005–10. doi: 10.1073/pnas.0508123103 16537476 10.1073/pnas.0508123103 PMC1389705

[28] Gunasekaran K, Pentony M, Shen M, Garrett L, Forte C, Woodward A, et al Enhancing antibody Fc heterodimer formation through electrostatic steering effects: applications to bispecific molecules and monovalent IgG. J Biol Chem. 2010 6 18; 285(25): 19637–46. doi: 10.1074/jbc.M110.117382 20400508 10.1074/jbc.M110.117382 PMC2885242

[29] Liu Z, Leng EC, Gunasekaran K, Pentony M, Shen M, Howard M, et al A novel antibody engineering strategy for making monovalent bispecific heterodimeric IgG antibodies by electrostatic steering mechanism. J Biol Chem. 2015 3 20; 290(12): 7535–62. doi: 10.1074/jbc.M114.620260 25583986 10.1074/jbc.M114.620260 PMC4367261

[30] Skegro D, Stutz C, Ollier R, Svensson E, Wassmann P, Florence B, et al Immunoglobulin domain interface exchange as a platform technology for the generation of Fc heterodimers and bispecific antibodies. J Biol Chem. 2017 4 27. 10.1074/jbc.M117.782433 PMC5465497 28450393

[31] Shields RL, Namenuk AK, Hong K, Meng YG, Rae J, Briggs J, et al High resolution mapping of the binding site on human IgG1 for Fc gamma RI, Fc gamma RII, Fc gamma RIII, and FcRn and design of IgG1 variants with improved binding to the Fc gamma R. J Biol Chem. 2001 3 2; 276(9): 6591–604. doi: 10.1074/jbc.M009483200 11096108 10.1074/jbc.M009483200

[32] Radaev S, Motyka S, Fridman WH, Sautes-Fridman C, Sun PD. The structure of a human type III Fcgamma receptor in complex with Fc. J Biol Chem. 2001 5 11; 276(19): 16469–77. doi: 10.1074/jbc.M100350200 11297532 10.1074/jbc.M100350200

[33] Kiyoshi M, Caaveiro JM, Kawai T, Tashiro S, Ide T, Asaoka Y, et al Structural basis for binding of human IgG1 to its high-affinity human receptor FcgammaRI. Nat Commun. 2015; 6: 6866 doi: 10.1038/ncomms7866 25925696 10.1038/ncomms7866 PMC4423232

[34] Saxena A, Wu D. Advances in Therapeutic Fc Engineering—Modulation of IgG-Associated Effector Functions and Serum Half-life. Front Immunol. 2016; 7: 580 doi: 10.3389/fimmu.2016.00580 28018347 10.3389/fimmu.2016.00580 PMC5149539

[35] Yan B, Boyd D, Kaschak T, Tsukuda J, Shen A, Lin Y, et al Engineering upper hinge improves stability and effector function of a human IgG1. J Biol Chem. 2012 2 17; 287(8): 5891–7. doi: 10.1074/jbc.M111.311811 22203673 10.1074/jbc.M111.311811 PMC3325591

[36] Grevys A, Bern M, Foss S, Bratlie DB, Moen A, Gunnarsen KS, et al Fc Engineering of Human IgG1 for Altered Binding to the Neonatal Fc Receptor Affects Fc Effector Functions. J Immunol. 2015 6 01; 194(11): 5497–508. doi: 10.4049/jimmunol.1401218 25904551 10.4049/jimmunol.1401218 PMC4432726

[37] Burmeister WP, Gastinel LN, Simister NE, Blum ML, Bjorkman PJ. Crystal structure at 2.2 A resolution of the MHC-related neonatal Fc receptor. Nature. 1994 11 24; 372(6504): 336–43. doi: 10.1038/372336a0 7969491 10.1038/372336a0

[38] Vaughn DE, Bjorkman PJ. Structural basis of pH-dependent antibody binding by the neonatal Fc receptor. Structure. 1998 1 15; 6(1): 63–73. 9493268 10.1016/s0969-2126(98)00008-2

[39] Hu S, Shively L, Raubitschek A, Sherman M, Williams LE, Wong JY, et al Minibody: A novel engineered anti-carcinoembryonic antigen antibody fragment (single-chain Fv-CH3) which exhibits rapid, high-level targeting of xenografts. Cancer Res. 1996 7 1; 56(13): 3055–61. 8674062

[40] Schaefer W, Regula JT, Bahner M, Schanzer J, Croasdale R, Durr H, et al Immunoglobulin domain crossover as a generic approach for the production of bispecific IgG antibodies. Proc Natl Acad Sci U S A. 2011 7 05; 108(27): 11187–92. doi: 10.1073/pnas.1019002108 21690412 10.1073/pnas.1019002108 PMC3131342

[41] Merchant AM, Zhu Z, Yuan JQ, Goddard A, Adams CW, Presta LG, et al An efficient route to human bispecific IgG. Nat Biotechnol. 1998 7; 16(7): 677–81. doi: 10.1038/nbt0798-677 9661204 10.1038/nbt0798-677

[42] Krah S, Schroter C, Eller C, Rhiel L, Rasche N, Beck J, et al Generation of human bispecific common light chain antibodies by combining animal immunization and yeast display. Protein Eng Des Sel. 2017 4 01; 30(4): 291–301. doi: 10.1093/protein/gzw077 28062646 10.1093/protein/gzw077

[43] Moore PA, Zhang W, Rainey GJ, Burke S, Li H, Huang L, et al Application of dual affinity retargeting molecules to achieve optimal redirected T-cell killing of B-cell lymphoma. Blood. 2011 4 28; 117(17): 4542–51. doi: 10.1182/blood-2010-09-306449 21300981 10.1182/blood-2010-09-306449

