# Supplementary figures and images for: An antibody with Fab-constant domains exchanged for a pair of CH3 domains
Source: PLoS One. 2018 Apr 9;13(4):e0195442. doi: 10.1371/journal.pone.0195442 (PMC5891013; doi:10.1371/journal.pone.0195442)

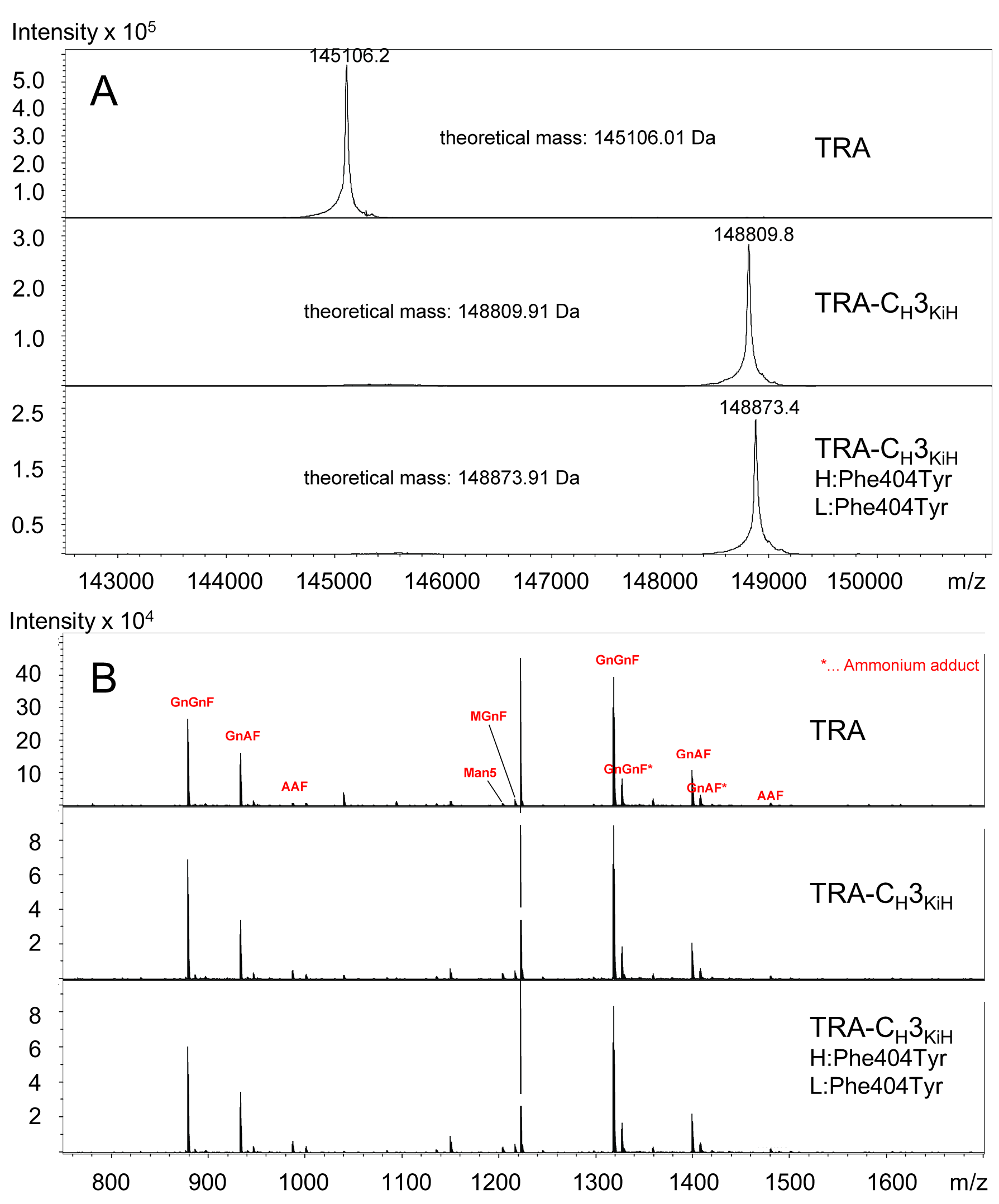

Supplement: S1 Fig — (A) intact mass analysis of TRA, TRA-CH3KiH and TRA-CH3KiH H:Phe404Tyr//L:Phe404Tyr, (B) glycan pattern analysis of trastuzumab and the domain exchanged antibody variants. The peak heights in the MS spectra roughly reflect the molar ratios of the glycoforms (note that more than one charge state is present per glycoform). (TIF) [file pone.0195442.s001.tif]

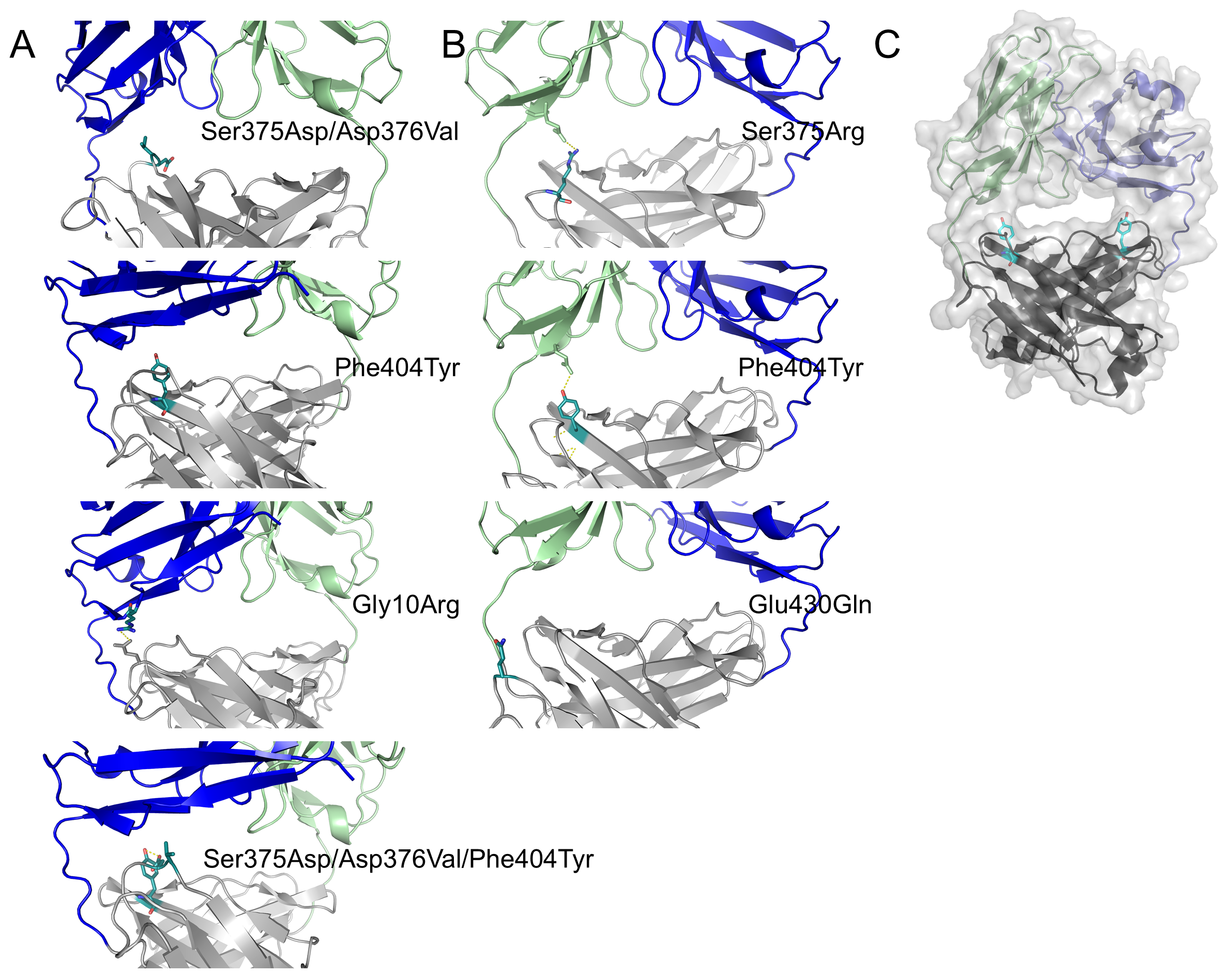

Supplement: S2 Fig — (A) interface mutations in the heavy chain, (B) interface mutations in the light chain, (C) surfaced cartoon diagram of TRA-CH3KiH H:Phe404Tyr//L:Phe404Tyr. VH: blue, Vκ: green, CH3: gray, mutated residues: teal. (TIF) [file pone.0195442.s002.tif]

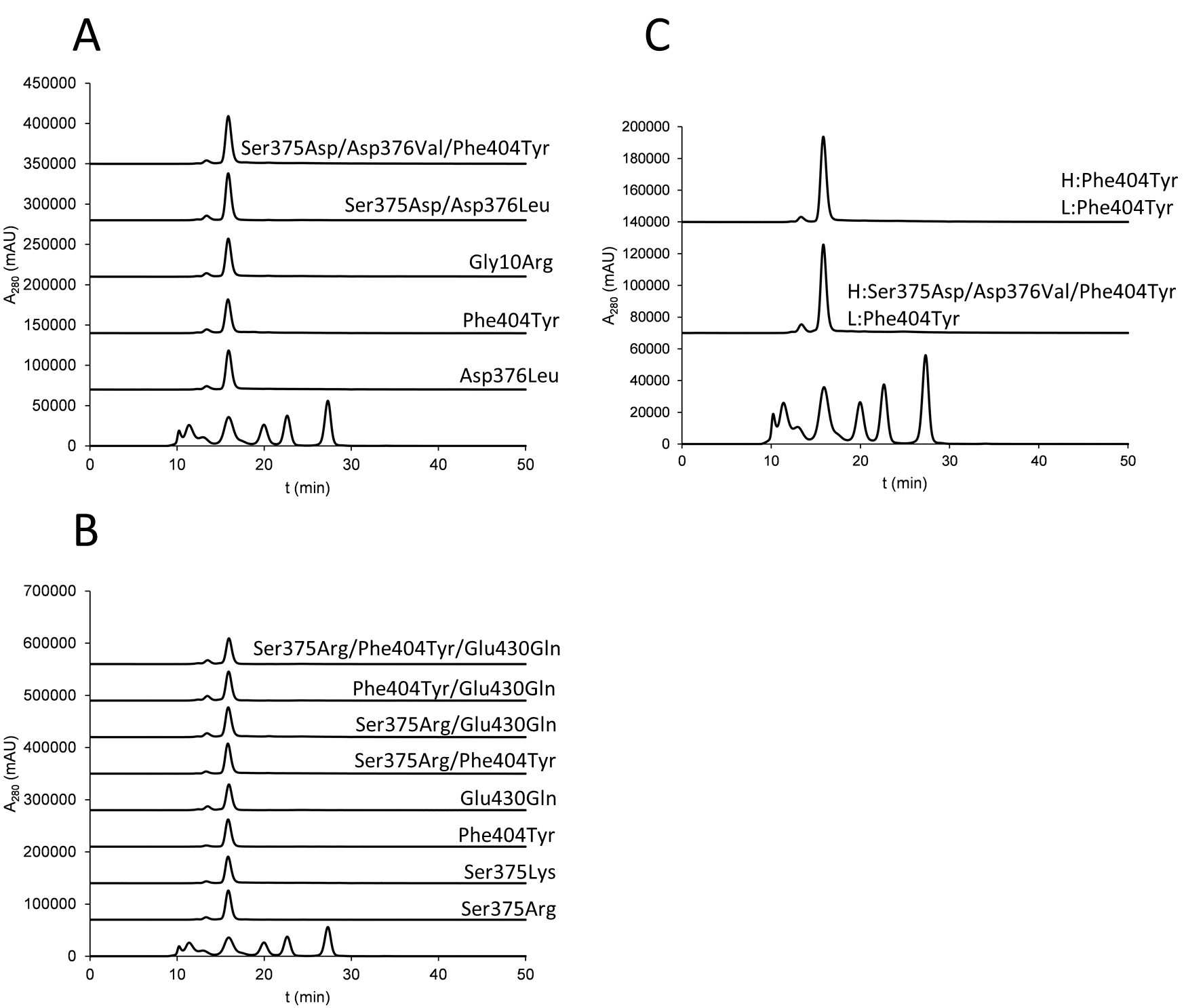

Supplement: S3 Fig — (A) interface mutants modified in the heavy chain, (B) interface mutants modified in the light chain, (C) interface mutants modified in both chains. (TIF) [file pone.0195442.s003.tif]

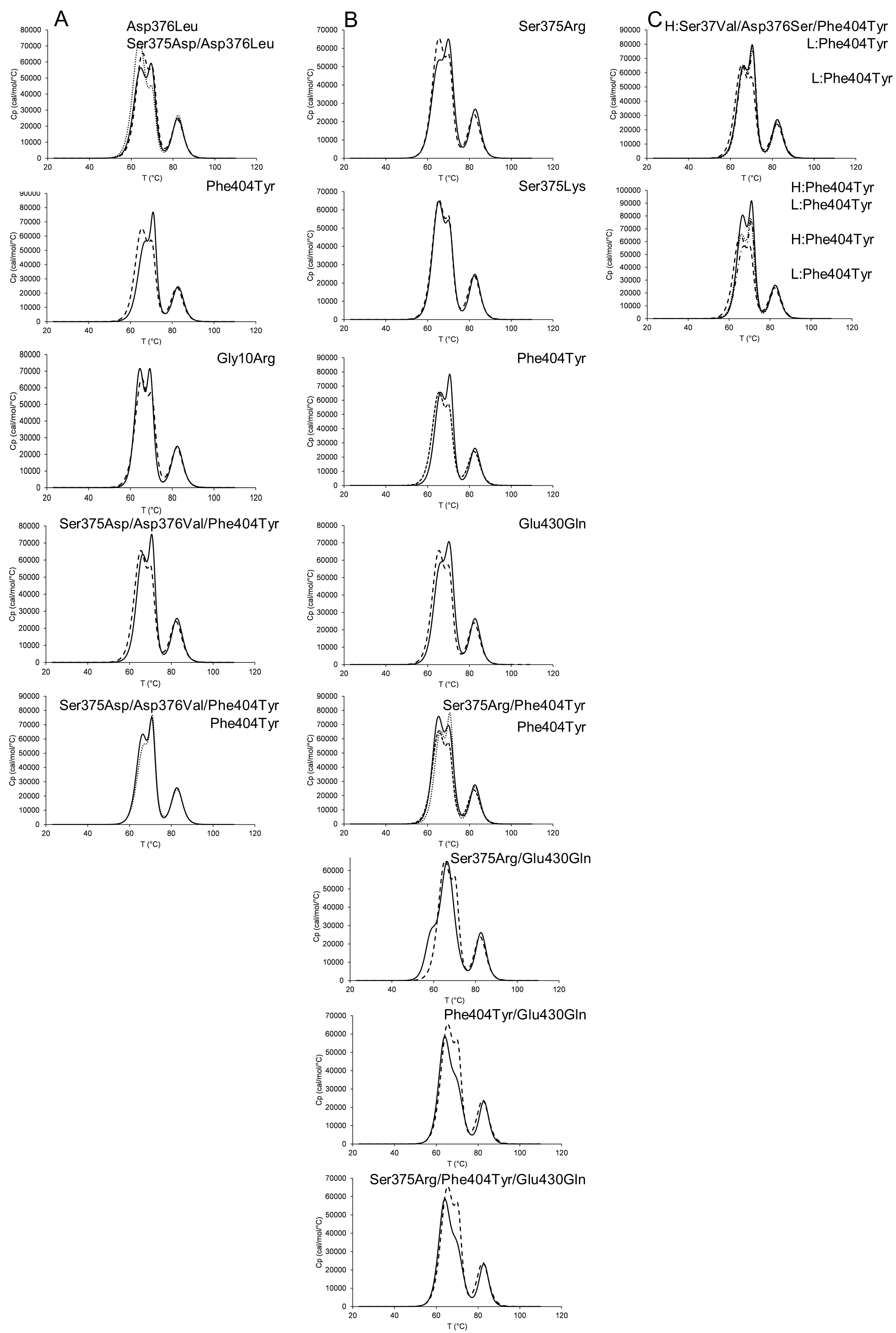

Supplement: S4 Fig — (A) thermograms of interface mutants modified in the heavy chain, (B) thermograms of interface mutants modified in the light chain, (C) thermograms of the interface mutants modified in both chains. Thermogram of the parental construct TRA-CH3KiH is depicted with dashed line and thermograms of the mutants with full line. Where the comparison to other mutants is shown, the thermogram of TRA-CH3KiH L:Phe404Tyr is depicted with a dotted line and the thermogram of TRA-CH3KiH H:Phe404Tyr with a dash-dot-dash line. (TIF) [file pone.0195442.s004.tif]
