# Supplementary material for: An antibody with Fab-constant domains exchanged for a pair of CH3 domains
Source: PLoS One. 2018 Apr 9;13(4):e0195442. doi: 10.1371/journal.pone.0195442 (PMC5891013; doi:10.1371/journal.pone.0195442)
Supplement: S1 Table — The lettering is in the color code corresponding to the construct schematic in Fig 1. Mutations driving the heterodimerization are in red. (DOCX) [file pone.0195442.s005.docx]

| **TRA-C_H_3_KiH_** |
| --- |
| Heavy chain (V_H_-C_H_3_H_-C_H_2-C_H_3) |
| **EVQLVESGGGLVQPGGSLRLSCAASGFNIKDTYIHWVRQAPGKGLEWVARIYPTNGYTRYADSVKGRFTISADTSKNTAYLQMNSLRAEDTAVYYCSRWGGDGFYAMDYWGQGTLVTVSSASTKGEPQVYTLPPSRDELTKNQVSLYCLVKGFYPSDIAVEWESNGQPENNYKTTPPVLDSDGSFFLYSKLTVDKSRWQQGNVFSCSVMHEALHNHYTQKSLSLSKSCDKTHTCPPCPAPELLGGPSVFLFPPKPKDTLMISRTPEVTCVVVDVSHEDPEVKFNWYVDGVEVHNAKTKPREEQYNSTYRVVSVLTVLHQDWLNGKEYKCKVSNKALPAPIEKTISKAKGQPREPQVYTLPPSRDELTKNQVSLTCLVKGFYPSDIAVEWESNGQPENNYKTTPPVLDSDGSFFLYSKLTVDKSRWQQGNVFSCSVMHEALHNHYTQKSLSLSPGK** |
| Light chain (V_κ_-C_H_3_κ_) |
| **DIQMTQSPSSLSASVGDRVTITCRASQDVNTAVAWYQQKPGKAPKLLIYSASFLYSGVPSRFSGSRSGTDFTLTISSLQPEDFATYYCQQHYTTPPTFGQGTKVEIKRTVAEPQVYTLPPSRDELTKNQVSLTCLVKGFYPSDIAVEWESNGQPENNYKTTPPVLDSDGSFFLTSKLTVDKSRWQQGNVFSCSVMHEALHNHYTQKSLSLSGEC** |

| **TRA-C_H_3_ZW1_** |
| --- |
| Heavy chain (V_H_-C_H_3_H_-C_H_2-C_H_3) |
| **EVQLVESGGGLVQPGGSLRLSCAASGFNIKDTYIHWVRQAPGKGLEWVARIYPTNGYTRYADSVKGRFTISADTSKNTAYLQMNSLRAEDTAVYYCSRWGGDGFYAMDYWGQGTLVTVSSASTKGEPQVYVLPPSRDELTKNQVSLLCLVKGFYPSDIAVEWESNGQPENNYLTWPPVLDSDGSFFLYSKLTVDKSRWQQGNVFSCSVMHEALHNHYTQKSLSLSKSCDKTHTCPPCPAPELLGGPSVFLFPPKPKDTLMISRTPEVTCVVVDVSHEDPEVKFNWYVDGVEVHNAKTKPREEQYNSTYRVVSVLTVLHQDWLNGKEYKCKVSNKALPAPIEKTISKAKGQPREPQVYTLPPSRDELTKNQVSLTCLVKGFYPSDIAVEWESNGQPENNYKTTPPVLDSDGSFFLYSKLTVDKSRWQQGNVFSCSVMHEALHNHYTQKSLSLSPGK** |
| Light chain (V_κ_-C_H_3_κ_) |
| **DIQMTQSPSSLSASVGDRVTITCRASQDVNTAVAWYQQKPGKAPKLLIYSASFLYSGVPSRFSGSRSGTDFTLTISSLQPEDFATYYCQQHYTTPPTFGQGTKVEIKRTVAEPQVYVYLPPSRDELTKNQVSLTCLVKGFYPSDIAVEWESNGQPENNYKTTPPVLDSDGSFALVSKLTVDKSRWQQGNVFSCSVMHEALHNHYTQKSLSLSGEC** |
