# Supplementary material for: An antibody with Fab-constant domains exchanged for a pair of CH3 domains
Source: PLoS One. 2018 Apr 9;13(4):e0195442. doi: 10.1371/journal.pone.0195442 (PMC5891013; doi:10.1371/journal.pone.0195442)
Supplement: S2 Table — Melting points of the mutants as determined with DSC are given in bold and are average of at least three experiments. (DOCX) [file pone.0195442.s006.docx]

|  | | | | | | | | |  | |  |  |
| --- | --- | --- | --- | --- | --- | --- | --- | --- | --- | --- | --- | --- |
| Construct | |  | |  | | | | | Melting temperatures (°C) * | | | |
|  | |  | |  | | | | | Tm1 | | Tm2 | Tm3 |
| Trastuzumab | |  | |  | | | | | **72.60±0.26** | | **80.69±0.20** |  |
|  | |  | |  | | | | | 72.97 | | 80.97 |  |
|  | |  | |  | | | | | 72.48 | | 80.53 |  |
|  | |  | |  | | | | | 72.36 | | 80.58 |  |
| TRA-C_H_3_KiH_ | |  | |  | | | | | **65.25±0.09** | | **70.30±0.07** | **81.95±0.01** |
|  | |  | |  | | | | | 65.21 | | 70.27 | 81.96 |
|  | |  | |  | | | | | 65.17 | | 70.24 | 81.96 |
|  | |  | |  | | | | | 65.38 | | 70.40 | 81.94 |
| TRA-Fab | |  | |  | | | | | **81.87±0.13** | |  |  |
|  | |  | |  | | | | | 81.91 | |  |  |
|  | |  | |  | | | | | 81.70 | |  |  |
|  | |  | |  | | | | | 82.00 | |  |  |
| TRA-FabC_H_3_KiH_ | |  | |  | | | | | **66.58±0.10** | |  |  |
|  | |  | |  | | | | | 66.71 | |  |  |
|  | |  | |  | | | | | 66.50 | |  |  |
|  | |  | |  | | | | | 66.52 | |  |  |
| TRA-Fab-C_H_3_ZW1_ | |  | |  | | | | | **71.08±0.25** | | **86.87±0.54** |  |
|  | |  | |  | | | | | 71.43 | | 87.63 |  |
|  | |  | |  | | | | | 70.91 | | 86.49 |  |
|  | |  | |  | | | | | 70.91 | | 86.49 |  |
| Mutants of TRA-C_H_3_KiH_ | | |  | | | | |  |  | |  |  |
|  | | Mutation | | Rationale | | | | | |  | | |
| Heavy chain (V_H_-C_H_3_H_) | | | | | | | | |  | |  |  |
| Single mutations | | | | | | | | | Tm1 | | Tm2 | Tm3 |
| BC-loop | | Asp376Leu | | increase hydrophobic packing of interface | | | | | **63.90±0.01** | | **70.14±0.00** | **82.45±0.01** |
|  | |  | |  | | | | | 63.90 | | 70.14 | 82.46 |
|  | |  | |  | | | | | 63.89 | | 70.14 | 82.43 |
|  | |  | |  | | | | | 63.90 | | 70.14 | 82.46 |
| DE-loop | | Phe404Tyr | | decrease hydrophobicity, H-bond to Asp375 | | | | | **66.56±0.18** | | **70.67±0.29** | **82.81±0.01** |
|  | |  | |  | | | | | 66.73 | | 71.07 | 82.80 |
|  | |  | |  | | | | | 66.73 | | 70.78 | 82.80 |
|  | |  | |  | | | | | 66.39 | | 70.27 | 82.81 |
|  | |  | |  | | | | | 66.37 | | 70.57 | 82.81 |
| FG-loop | | Gly10Arg (V_H_) | | salt bridge to Glu430 | | | | | **64.23±0.16** | | **69.33±0.25** | **82.74±0.26** |
|  | |  | |  | | | | | 64.41 | | 69.57 | 82.43 |
|  | |  | |  | | | | | 64.36 | | 69.60 | 82.52 |
|  | |  | |  | | | | | 64.03 | | 69.09 | 83.04 |
|  | |  | |  | | | | | 64.12 | | 69.07 | 82.95 |
| Combined mutations | | | | | | | | | | |  |  |
|  | | Ser375Asp/Asp376Leu | | | |  | | | **64.40±0.12** | | **69.76±0.19** | **82.60±0.39** |
|  | |  | | | |  | | | 64.31 | | 69.90 | 82.33 |
|  | |  | | | |  | | | 64.32 | | 69.90 | 82.32 |
|  | |  | | | |  | | | 64.58 | | 69.49 | 83.16 |
|  | | Ser375Asp/Asp376Val/Phe404Tyr | | | |  | | | **65.99±0.17** | | **70.69±0.29** | **82.70±0.01** |
|  | |  | | | |  | | | 66.12 | | 70.90 | 82.70 |
|  | |  | | | |  | | | 66.10 | | 70.89 | 82.71 |
|  | |  | | | |  | | | 65.74 | | 70.27 | 82.69 |
| Light chain (V_κ_-C_H_3_κ_) | | | | | | | | | | |  |  |
| Single mutations | | | | | | | | | | |  |  |
| BC-loop | | Ser375Arg | | salt bridge to Glu105 (V_κ_) | | | | | **65.36±0.47** | | **70.10±0.31** | **82.83±0.05** |
|  | |  | |  | | | | | 64.91 | | 70.39 | 82.80 |
|  | |  | |  | | | | | 64.88 | | 70.43 | 82.76 |
|  | |  | |  | | | | | 65.82 | | 69.80 | 82.85 |
|  | |  | |  | | | | | 65.83 | | 69.79 | 82.89 |
|  | | Ser375Lys | | salt bridge to Glu105 (V_κ_) | | | | | **64.98±0.23** | | **70.22±0.38** | **82.75±0.11** |
|  | |  | |  | | | | | 65.20 | | 70.60 | 82.64 |
|  | |  | |  | | | | | 64.76 | | 69.85 | 82.65 |
|  | |  | |  | | | | | 64.74 | | 69.84 | 82.86 |
|  | |  | |  | | | | | 65.21 | | 70.59 | 82.86 |
| DE-loop | | Phe404Tyr | | decrease hydrophobicity, H-bond to Glu105 (V_κ_) | | | | | **66.01±0.06** | | **70.63±0.13** | **82.77±0.07** |
|  | |  | |  | | | | | 66.05 | | 70.78 | 82.68 |
|  | |  | |  | | | | | 66.08 | | 70.73 | 82.73 |
|  | |  | |  | | | | | 65.95 | | 70.48 | 82.85 |
|  | |  | |  | | | | | 65.95 | | 70.53 | 82.80 |
| FG-loop | | Glu430Gln | | remove unbalanced charge | | | | | **66.01±0.36** | | **70.49±0.19** | **82.87±0.18** |
|  | |  | |  | | | | | 65.64 | | 70.67 | 82.68 |
|  | |  | |  | | | | | 65.66 | | 70.68 | 82.70 |
|  | |  | |  | | | | | 66.38 | | 70.30 | 83.05 |
|  | |  | |  | | | | | 66.37 | | 70.30 | 83.04 |
| Combined mutations | | | | | | | | | | |  |  |
|  | | Ser375Arg/Phe404Tyr | | |  | | | | **64.78±0.30** | | **70.30±0.24** | **82.72±0.10** |
|  | |  | | |  | | | | 64.58 | | 70.53 | 82.68 |
|  | |  | | |  | | | | 64.55 | | 70.39 | 82.63 |
|  | |  | | |  | | | | 65.20 | | 69.97 | 82.86 |
|  | | Ser375Arg/Glu430Gln | | |  | | | | **64.11±0.58** | | **69.82±0.31** | **82.81±0.15** |
|  | |  | | |  | | | | 64.41 | | 70.04 | 82.93 |
|  | |  | | |  | | | | 64.62 | | 70.04 | 82.90 |
|  | |  | | |  | | | | 63.30 | | 69.39 | 82.60 |
|  | | Phe404Tyr/Glu430Gln | | |  | | | | **59.38±0.81** | | **66.25±0.31** | **82.63±0.02** |
|  | |  | | |  | | | | 58.59 | | 66.54 | 82.66 |
|  | |  | | |  | | | | 58.56 | | 66.59 | 82.63 |
|  | |  | | |  | | | | 60.17 | | 65.95 | 82.60 |
|  | |  | | |  | | | | 60.22 | | 65.93 | 82.63 |
|  | | Ser375Arg/Phe404Tyr/Glu430Gln | | | | | | | **63.95±0.07** | | **69.55±0.55** | **82.95±0.13** |
|  | |  | | | | | | | 63.89 | | 70.09 | 82.20 |
|  | |  | | | | | | | 64.01 | | 69.00 | 83.10 |
|  | |  | | | | | | | 64.02 | | 68.99 | 83.05 |
|  | |  | | | | | | | 63.87 | | 70.11 | 82.85 |
| Heavy/light chain |  | | | |  | | | |  | |  |  |
|  | | H:Phe404Tyr//L:Phe404Tyr | | | | |  | | **66.57±0.20** | | **71.06±0.19** | **82.71±0.23** |
|  | |  | | | | |  | | 66.84 | | 71.20 | 82.54 |
|  | |  | | | | |  | | 66.47 | | 71.19 | 82.56 |
|  | |  | | | | |  | | 66.39 | | 70.80 | 83.04 |
|  | | H:Ser375Asp/Asp376Val/Phe404Tyr//L:Phe404Tyr | | | | | | | **65.89±0.17** | | **70.43±0.27** | **82.69±0.26** |
|  | |  | | | | | | | 66.06 | | 70.69 | 82.45 |
|  | |  | | | | | | | 66.05 | | 70.71 | 82.42 |
|  | |  | | | | | | | 65.74 | | 70.15 | 82.93 |
|  | |  | | | | | | | 65.70 | | 70.16 | 82.97 |

- For thermogram overlays and comparison with the unmodified TRA-C_H_3_KiH_ please see S4 Fig.
