# Supplementary material for: An antibody with Fab-constant domains exchanged for a pair of CH3 domains
Source: PLoS One. 2018 Apr 9;13(4):e0195442. doi: 10.1371/journal.pone.0195442 (PMC5891013; doi:10.1371/journal.pone.0195442)
Supplement: S3 Table — (DOCX) [file pone.0195442.s007.docx]

| Heterodimerization  mutations |  | Forward primer | Reverse primer |
| --- | --- | --- | --- |
| **TRA-C_H_3_KiH_** |  |  |  |
| V_H_-C_H_3_H_ |  |  |  |
|  | Thr366Tyr | GAACCAGGTCAGCCTGTACTGCCTGGTCAAAGGCTTC | GAAGCCTTTGACCAGGCAGTACAGGCTGACCTGGTTC |
| V_κ_-C_H_3_κ_ |  |  |  |
|  | Tyr407Thr | GACGGCTCCTTCTTCCTCACTAGCAAGCTCACCGTG | CACGGTGAGCTTGCTAGTGAGGAAGAAGGAGCCGTC |
| **TRA-C_H_3_ZW1_** |  |  |  |
| V_H_-C_H_3_H_ |  |  |  |
|  | Thr350Val | GAACCACAGGTGTACGTGCTGCCCCCATCCCGG | CCGGGATGGGGGCAGCACGTACACCTGTGGTTC |
|  | Thr366Leu | GAACCAGGTCAGCCTGTTGTGCCTGGTCAAAGGCTTC | GAAGCCTTTGACCAGGCACAACAGGCTGACCTGGTTC |
|  | Lys392Leu/Thr394Trp | CCGGAGAACAACTACCTTACCTGGCCTCCCGTGCTGGAC | GTCCAGCACGGGAGGCCAGGTAAGGTAGTTGTTCTCCGG |
| V_κ_-C_H_3_κ_ |  |  |  |
|  | Thr350Val/Leu351Tyr | GAACCACAGGTGTACGTGTATCCCCCATCCCGGGATGAG | CTCATCCCGGGATGGGGGATACACGTACACCTGTGGTTC |
|  | Phe405Ala/Tyr407Val | TCCGACGGCTCCTTCGCGCTCGTCAGCAAGCTCACCGTG | CACGGTGAGCTTGCTGACGAGCGCGAAGGAGCCGTCGGA |
| Interface mutations |  |  |  |
| **TRA-C_H_3_KiH_** |  |  |  |
| V_H_-C_H_3_H_ |  |  |  |
|  | Ser375Asp/Asp376Val | CAAAGGCTTCTATCCCGACGTTATCGCCGTGGAGTGG | CCACTCCACGGCGATAACGTCGGGATAGAAGCCTTTG |
|  | Phe404Tyr | CTGGACTCCGACGGCTCCTACTTCCTCTACAGCAAGCTCAC | GTGAGCTTGCTGTAGAGGAAGTAGGAGCCGTCGGAGTCCAG |
|  | Gly10Arg | GTGGAGTCTGGCGGTAGGCTGGTGCAGCCAGGGGGCTC | GAGCCCCCTGGCTGCACCAGCCTACCGCCAGACTCCAC |
| V_κ_-C_H_3_κ_ |  |  |  |
|  | Ser375Arg | CAAAGGCTTCTATCCCCGAGACATCGCCGTGGAGTGG | CCACTCCACGGCGATGTCTCGGGGATAGAAGCCTTTG |
|  | Phe404Tyr | CTGGACTCCGACGGCTCCTACTTCCTCACTAGCAAGCTCAC | GTGAGCTTGCTAGTGAGGAAGTAGGAGCCGTCGGAGTCCAG |
|  | Gln430Glu | CTCATGCTCCGTGATGCATCAGGCTCTGCACAACCACTAC | GTAGTGGTTGTGCAGAGCCTGATGCATCACGGAGCATGAG |
| **TRA-C_H_3_ZW1_ L:Phe404Tyr** |  |  |  |
| V_κ_-C_H_3_κ_ |  |  |  |
|  | Phe404Tyr | TCCGACGGCTCCTACGCGCTCGTCAGCAAGCTCACCGTG | CACGGTGAGCTTGCTGACGAGCGCGTAGGAGCCGTCGGA |
