# Supplementary material for: An antibody with Fab-constant domains exchanged for a pair of CH3 domains
Source: PLoS One. 2018 Apr 9;13(4):e0195442. doi: 10.1371/journal.pone.0195442 (PMC5891013; doi:10.1371/journal.pone.0195442)
Supplement: S4 Table — The theoretical mass is calculated by the summation of all chains (2*HC + 2*LC), minus the number of cysteine bridges multiplied by two (-32, in the theoretical mass cysteines are reduced–so the hydrogen mass has to be subtracted), plus the number of glycosylation sites (+2, deglycosylation will lead to N ➔ D conversion) and +1 for the charge. The theoretical masses were calculated using the online tool peptide mass (http://web.expasy.org/peptide_mass/). (DOCX) [file pone.0195442.s008.docx]

| Protein | Molecular weight (Da) |
| --- | --- |
| Trastuzumab |  |
| Heavy chain | 49124.41 |
| Light chain | 23443.10 |
| Theoretical molecular weight | 145106.01 |
| TRA-C_H_3_KiH_ |  |
| Heavy chain | 50730.03 |
| Light chain | 23689.43 |
| Theoretical molecular weight | 148809.91 |
| TRA-C_H_3_KiH_ H:Phe404Tyr//L:Phe404Tyr |  |
| Heavy chain | 50746.03 |
| Light chain | 23705.42 |
| Theoretical molecular weight | 148873.91 |
