# Supplementary material for: An antibody with Fab-constant domains exchanged for a pair of CH3 domains
Source: PLoS One. 2018 Apr 9;13(4):e0195442. doi: 10.1371/journal.pone.0195442 (PMC5891013; doi:10.1371/journal.pone.0195442)
Supplement: S5 Table — Proportion of glycoforms in % including the non-glycosylated peptide. The proglycan nomenclature (http://www.proglycan.com/protein-glycosylation-analysis/nomenclature) was used and only one possible isomer is annotated. (DOCX) [file pone.0195442.s009.docx]

| Glycan |  | Trastuzumab | TRA-CH3KiH | TRA-CH3KiH H:Phe404Tyr//L:Phe404/Tyr |
| --- | --- | --- | --- | --- |
| not glycosylated |  | 0.76 | 0.58 | 0.59 |
| Man5 |  | 0.68 | 1.22 | 1.16 |
| GnGn | G0 | 0.41 | 0.73 | 0.70 |
| MGnF |  | 1.88 | 2.04 | 2.11 |
| GnGnF | G0F | 61.06 | 63.60 | 61.29 |
| GnAF | G1F | 28.28 | 24.02 | 25.25 |
| AAF | G2F | 2.99 | 3.50 | 4.31 |
| NaAF |  | 0.20 | 0.34 | 0.53 |
| NaNaF |  | 0.07 | 0.14 | 0.12 |
| GnGn(bi) | G0bF | 2.03 | 2.22 | 1.98 |
| GnA(bi) | G1bF | 1.33 | 1.37 | 1.64 |
| AA(bi) | G2bF | 0.30 | 0.24 | 0.32 |
